# Supplementary material for: Utility of Same-Modality, Cross-Domain Transfer Learning for Malignant Bone Tumor Detection on Radiographs: A Multi-Faceted Performance Comparison with a Scratch-Trained Model
Source: Cancers (Basel). 2025 Sep 27;17(19):3144. doi: 10.3390/cancers17193144 (PMC12523960; doi:10.3390/cancers17193144)
Supplement: Supplementary file 1 [file cancers-17-03144-s001.zip › Figure S1.pdf]

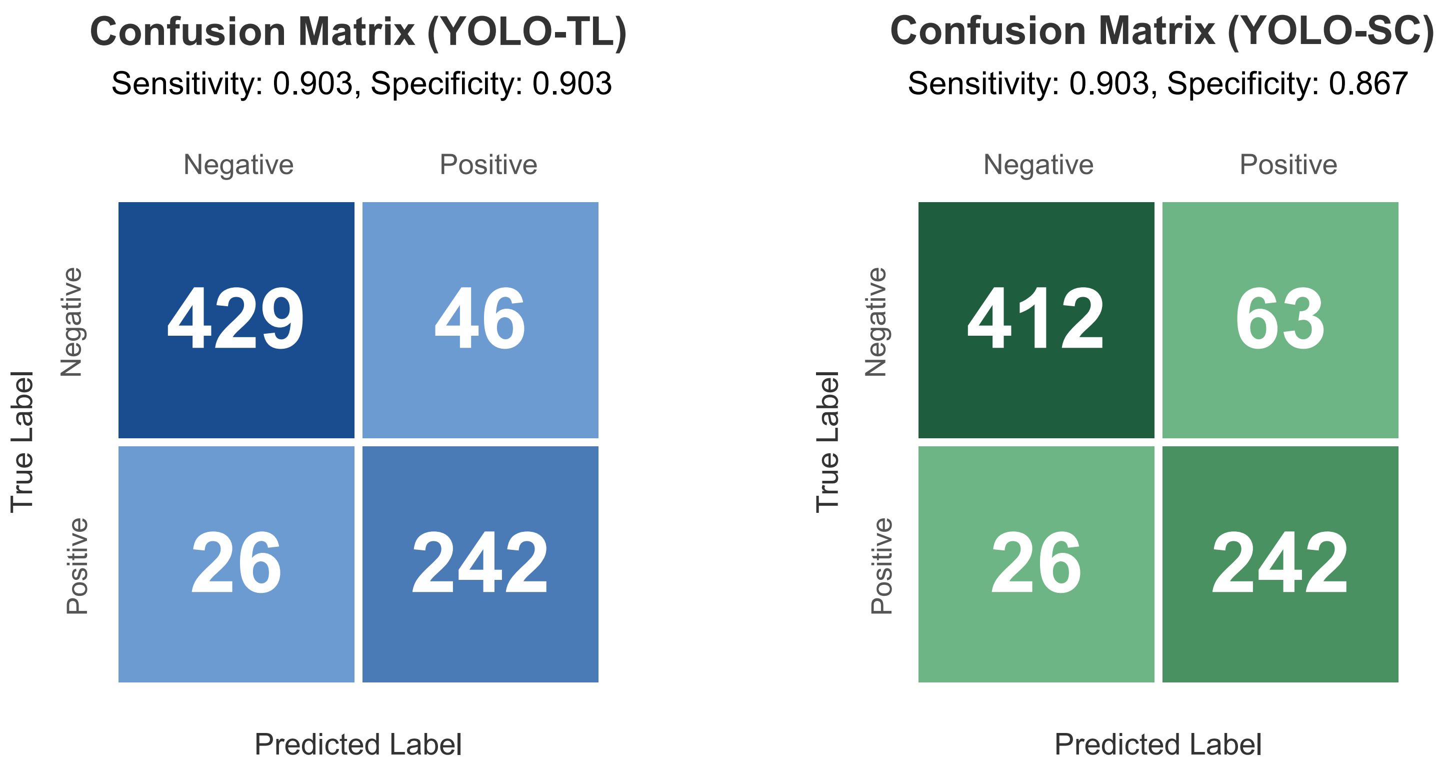

Confusion Matrix (YOLO-TL)
Sensitivity: 0.903, Specificity: 0.903
Negative
Positive
True Label
Negative
Positive
429
46
26
242
Predicted Label
Confusion Matrix (YOLO-SC)
Sensitivity: 0.903, Specificity: 0.867
Negative
Positive
True Label
Negative
Positive
412
63
26
242
Predicted Label

Figure S1
