# Supplementary material for: Utility of Same-Modality, Cross-Domain Transfer Learning for Malignant Bone Tumor Detection on Radiographs: A Multi-Faceted Performance Comparison with a Scratch-Trained Model
Source: Cancers (Basel). 2025 Sep 27;17(19):3144. doi: 10.3390/cancers17193144 (PMC12523960; doi:10.3390/cancers17193144)
Supplement: Supplementary file 1 [file cancers-17-03144-s001.zip › Table S1. YOLO-SC.pdf]

filename	label	score
../data/osakakokusai/oosaka_pos/517.dcm	1	0.012146
../data/osakakokusai/oosaka_pos/462.dcm	1	0.03754
../data/osakakokusai/oosaka_pos/477.dcm	1	0.0542
../data/osakakokusai/oosaka_pos/483.dcm	1	0.06076
../data/osakakokusai/oosaka_pos/241.dcm	1	0.06537
../data/osakakokusai/oosaka_pos/137.dcm	1	0.0677
../data/osakakokusai/oosaka_pos/238.dcm	1	0.0716
../data/osakakokusai/oosaka_pos/463.dcm	1	0.0742
../data/osakakokusai/oosaka_pos/459.dcm	1	0.08124
../data/osakakokusai/oosaka_pos/345.dcm	1	0.0854
../data/osakakokusai/oosaka_pos/456.dcm	1	0.0874
../data/osakakokusai/oosaka_pos/425.dcm	1	0.0888
../data/osakakokusai/oosaka_pos/239.dcm	1	0.0981
../data/osakakokusai/oosaka_pos/341.dcm	1	0.1
../data/osakakokusai/oosaka_pos/237.dcm	1	0.10614
../data/osakakokusai/oosaka_pos/426.dcm	1	0.118
../data/osakakokusai/oosaka_pos/445.dcm	1	0.1432
../data/osakakokusai/oosaka_pos/32.dcm	1	0.1571
../data/osakakokusai/oosaka_pos/476.dcm	1	0.1577
../data/osakakokusai/oosaka_pos/148.dcm	1	0.1592
../data/osakakokusai/oosaka_pos/352.dcm	1	0.181
../data/osakakokusai/oosaka_pos/58.dcm	1	0.205
../data/osakakokusai/oosaka_pos/135.dcm	1	0.205
../data/osakakokusai/oosaka_pos/547.dcm	1	0.2089
../data/osakakokusai/oosaka_pos/72.dcm	1	0.2114
../data/osakakokusai/oosaka_pos/73.dcm	1	0.2186
../data/osakakokusai/oosaka_pos/416.dcm	1	0.223
../data/osakakokusai/oosaka_pos/469.dcm	1	0.2299
../data/osakakokusai/oosaka_pos/423.dcm	1	0.2415
../data/osakakokusai/oosaka_pos/150.dcm	1	0.2568
../data/osakakokusai/oosaka_pos/518.dcm	1	0.2603
../data/osakakokusai/oosaka_pos/455.dcm	1	0.2617
../data/osakakokusai/oosaka_pos/87.dcm	1	0.2783
../data/osakakokusai/oosaka_pos/441.dcm	1	0.2817
../data/osakakokusai/oosaka_pos/480.dcm	1	0.2847
../data/osakakokusai/oosaka_pos/457.dcm	1	0.2903
../data/osakakokusai/oosaka_pos/417.dcm	1	0.299
../data/osakakokusai/oosaka_pos/145.dcm	1	0.3003
../data/osakakokusai/oosaka_pos/107.dcm	1	0.3052

../data/osakakokusai/oosaka_pos/89.dcm	1	0.3254
../data/osakakokusai/oosaka_pos/90.dcm	1	0.3306
../data/osakakokusai/oosaka_pos/147.dcm	1	0.3386
../data/osakakokusai/oosaka_pos/293.dcm	1	0.3452
../data/osakakokusai/oosaka_pos/143.dcm	1	0.3455
../data/osakakokusai/oosaka_pos/464.dcm	1	0.3606
../data/osakakokusai/oosaka_pos/44.dcm	1	0.3638
../data/osakakokusai/oosaka_pos/71.dcm	1	0.372
../data/osakakokusai/oosaka_pos/236.dcm	1	0.3767
../data/osakakokusai/oosaka_pos/70.dcm	1	0.3872
../data/osakakokusai/oosaka_pos/94.dcm	1	0.3872
../data/osakakokusai/oosaka_pos/91.dcm	1	0.399
../data/osakakokusai/oosaka_pos/62.dcm	1	0.4082
../data/osakakokusai/oosaka_pos/323.dcm	1	0.4102
../data/osakakokusai/oosaka_pos/286.dcm	1	0.4216
../data/osakakokusai/oosaka_pos/160.dcm	1	0.433
../data/osakakokusai/oosaka_pos/339.dcm	1	0.435
../data/osakakokusai/oosaka_pos/105.dcm	1	0.4465
../data/osakakokusai/oosaka_pos/317.dcm	1	0.4534
../data/osakakokusai/oosaka_pos/422.dcm	1	0.4639
../data/osakakokusai/oosaka_pos/471.dcm	1	0.4766
../data/osakakokusai/oosaka_pos/447.dcm	1	0.4775
../data/osakakokusai/oosaka_pos/230.dcm	1	0.4824
../data/osakakokusai/oosaka_pos/475.dcm	1	0.4844
../data/osakakokusai/oosaka_pos/388.dcm	1	0.4863
../data/osakakokusai/oosaka_pos/240.dcm	1	0.4883
../data/osakakokusai/oosaka_pos/118.dcm	1	0.502
../data/osakakokusai/oosaka_pos/348.dcm	1	0.502
../data/osakakokusai/oosaka_pos/458.dcm	1	0.5156
../data/osakakokusai/oosaka_pos/424.dcm	1	0.5195
../data/osakakokusai/oosaka_pos/499.dcm	1	0.5254
../data/osakakokusai/oosaka_pos/88.dcm	1	0.5283
../data/osakakokusai/oosaka_pos/346.dcm	1	0.5303
../data/osakakokusai/oosaka_pos/93.dcm	1	0.5312
../data/osakakokusai/oosaka_pos/152.dcm	1	0.541
../data/osakakokusai/oosaka_pos/47.dcm	1	0.543
../data/osakakokusai/oosaka_pos/327.dcm	1	0.544
../data/osakakokusai/oosaka_pos/470.dcm	1	0.545
../data/osakakokusai/oosaka_pos/5.dcm	1	0.548
../data/osakakokusai/oosaka_pos/136.dcm	1	0.549

../data/osakakokusai/oosaka_pos/398.dcm	1	0.549
../data/osakakokusai/oosaka_pos/504.dcm	1	0.5537
../data/osakakokusai/oosaka_pos/460.dcm	1	0.5566
../data/osakakokusai/oosaka_pos/285.dcm	1	0.562
../data/osakakokusai/oosaka_pos/3.dcm	1	0.565
../data/osakakokusai/oosaka_pos/428.dcm	1	0.568
../data/osakakokusai/oosaka_pos/537.dcm	1	0.5737
../data/osakakokusai/oosaka_pos/430.dcm	1	0.5796
../data/osakakokusai/oosaka_pos/64.dcm	1	0.583
../data/osakakokusai/oosaka_pos/474.dcm	1	0.586
../data/osakakokusai/oosaka_pos/354.dcm	1	0.588
../data/osakakokusai/oosaka_pos/289.dcm	1	0.5938
../data/osakakokusai/oosaka_pos/13.dcm	1	0.596
../data/osakakokusai/oosaka_pos/85.dcm	1	0.6
../data/osakakokusai/oosaka_pos/319.dcm	1	0.6167
../data/osakakokusai/oosaka_pos/565.dcm	1	0.6167
../data/osakakokusai/oosaka_pos/303.dcm	1	0.6206
../data/osakakokusai/oosaka_pos/472.dcm	1	0.6206
../data/osakakokusai/oosaka_pos/103.dcm	1	0.626
../data/osakakokusai/oosaka_pos/473.dcm	1	0.626
../data/osakakokusai/oosaka_pos/539.dcm	1	0.63
../data/osakakokusai/oosaka_pos/297.dcm	1	0.6333
../data/osakakokusai/oosaka_pos/108.dcm	1	0.636
../data/osakakokusai/oosaka_pos/451.dcm	1	0.636
../data/osakakokusai/oosaka_pos/190.dcm	1	0.637
../data/osakakokusai/oosaka_pos/86.dcm	1	0.6377
../data/osakakokusai/oosaka_pos/186.dcm	1	0.6377
../data/osakakokusai/oosaka_pos/413.dcm	1	0.6416
../data/osakakokusai/oosaka_pos/79.dcm	1	0.644
../data/osakakokusai/oosaka_pos/207.dcm	1	0.66
../data/osakakokusai/oosaka_pos/144.dcm	1	0.662
../data/osakakokusai/oosaka_pos/74.dcm	1	0.6646
../data/osakakokusai/oosaka_pos/95.dcm	1	0.6646
../data/osakakokusai/oosaka_pos/211.dcm	1	0.6646
../data/osakakokusai/oosaka_pos/281.dcm	1	0.6655
../data/osakakokusai/oosaka_pos/535.dcm	1	0.6655
../data/osakakokusai/oosaka_pos/359.dcm	1	0.668
../data/osakakokusai/oosaka_pos/561.dcm	1	0.668
../data/osakakokusai/oosaka_pos/60.dcm	1	0.669
../data/osakakokusai/oosaka_pos/9.dcm	1	0.67

../data/osakakokusai/oosaka_pos/52.dcm	1	0.6724
../data/osakakokusai/oosaka_pos/349.dcm	1	0.675
../data/osakakokusai/oosaka_pos/295.dcm	1	0.676
../data/osakakokusai/oosaka_pos/122.dcm	1	0.677
../data/osakakokusai/oosaka_pos/134.dcm	1	0.6826
../data/osakakokusai/oosaka_pos/271.dcm	1	0.6836
../data/osakakokusai/oosaka_pos/329.dcm	1	0.6885
../data/osakakokusai/oosaka_pos/564.dcm	1	0.69
../data/osakakokusai/oosaka_pos/133.dcm	1	0.691
../data/osakakokusai/oosaka_pos/563.dcm	1	0.691
../data/osakakokusai/oosaka_pos/45.dcm	1	0.6934
../data/osakakokusai/oosaka_pos/209.dcm	1	0.6934
../data/osakakokusai/oosaka_pos/259.dcm	1	0.6934
../data/osakakokusai/oosaka_pos/11.dcm	1	0.6953
../data/osakakokusai/oosaka_pos/46.dcm	1	0.696
../data/osakakokusai/oosaka_pos/313.dcm	1	0.697
../data/osakakokusai/oosaka_pos/158.dcm	1	0.6978
../data/osakakokusai/oosaka_pos/229.dcm	1	0.698
../data/osakakokusai/oosaka_pos/406.dcm	1	0.699
../data/osakakokusai/oosaka_pos/49.dcm	1	0.703
../data/osakakokusai/oosaka_pos/503.dcm	1	0.7124
../data/osakakokusai/oosaka_pos/559.dcm	1	0.7124
../data/osakakokusai/oosaka_pos/540.dcm	1	0.713
../data/osakakokusai/oosaka_pos/205.dcm	1	0.714
../data/osakakokusai/oosaka_pos/7.dcm	1	0.7153
../data/osakakokusai/oosaka_pos/369.dcm	1	0.718
../data/osakakokusai/oosaka_pos/421.dcm	1	0.718
../data/osakakokusai/oosaka_pos/560.dcm	1	0.7188
../data/osakakokusai/oosaka_pos/48.dcm	1	0.7227
../data/osakakokusai/oosaka_pos/146.dcm	1	0.724
../data/osakakokusai/oosaka_pos/78.dcm	1	0.7266
../data/osakakokusai/oosaka_pos/101.dcm	1	0.7266
../data/osakakokusai/oosaka_pos/196.dcm	1	0.7285
../data/osakakokusai/oosaka_pos/310.dcm	1	0.7324
../data/osakakokusai/oosaka_pos/77.dcm	1	0.734
../data/osakakokusai/oosaka_pos/391.dcm	1	0.735
../data/osakakokusai/oosaka_pos/50.dcm	1	0.7363
../data/osakakokusai/oosaka_pos/192.dcm	1	0.7363
../data/osakakokusai/oosaka_pos/536.dcm	1	0.7363
../data/osakakokusai/oosaka_pos/99.dcm	1	0.739

../data/osakakokusai/oosaka_pos/309.dcm	1	0.7393
../data/osakakokusai/oosaka_pos/526.dcm	1	0.7393
../data/osakakokusai/oosaka_pos/533.dcm	1	0.74
../data/osakakokusai/oosaka_pos/178.dcm	1	0.7417
../data/osakakokusai/oosaka_pos/409.dcm	1	0.7417
../data/osakakokusai/oosaka_pos/449.dcm	1	0.743
../data/osakakokusai/oosaka_pos/246.dcm	1	0.744
../data/osakakokusai/oosaka_pos/43.dcm	1	0.7476
../data/osakakokusai/oosaka_pos/201.dcm	1	0.7476
../data/osakakokusai/oosaka_pos/400.dcm	1	0.7476
../data/osakakokusai/oosaka_pos/250.dcm	1	0.7485
../data/osakakokusai/oosaka_pos/395.dcm	1	0.7485
../data/osakakokusai/oosaka_pos/188.dcm	1	0.75
../data/osakakokusai/oosaka_pos/301.dcm	1	0.753
../data/osakakokusai/oosaka_pos/360.dcm	1	0.753
../data/osakakokusai/oosaka_pos/361.dcm	1	0.7534
../data/osakakokusai/oosaka_pos/482.dcm	1	0.755
../data/osakakokusai/oosaka_pos/251.dcm	1	0.757
../data/osakakokusai/oosaka_pos/538.dcm	1	0.757
../data/osakakokusai/oosaka_pos/36.dcm	1	0.758
../data/osakakokusai/oosaka_pos/131.dcm	1	0.7583
../data/osakakokusai/oosaka_pos/282.dcm	1	0.761
../data/osakakokusai/oosaka_pos/363.dcm	1	0.762
../data/osakakokusai/oosaka_pos/562.dcm	1	0.7627
../data/osakakokusai/oosaka_pos/173.dcm	1	0.764
../data/osakakokusai/oosaka_pos/199.dcm	1	0.764
../data/osakakokusai/oosaka_pos/396.dcm	1	0.7646
../data/osakakokusai/oosaka_pos/415.dcm	1	0.7676
../data/osakakokusai/oosaka_pos/120.dcm	1	0.769
../data/osakakokusai/oosaka_pos/393.dcm	1	0.7695
../data/osakakokusai/oosaka_pos/194.dcm	1	0.7705
../data/osakakokusai/oosaka_pos/392.dcm	1	0.771
../data/osakakokusai/oosaka_pos/365.dcm	1	0.7734
../data/osakakokusai/oosaka_pos/372.dcm	1	0.7734
../data/osakakokusai/oosaka_pos/544.dcm	1	0.7754
../data/osakakokusai/oosaka_pos/528.dcm	1	0.778
../data/osakakokusai/oosaka_pos/390.dcm	1	0.781
../data/osakakokusai/oosaka_pos/442.dcm	1	0.783
../data/osakakokusai/oosaka_pos/37.dcm	1	0.789
../data/osakakokusai/oosaka_pos/512.dcm	1	0.79

../data/osakakokusai/oosaka_pos/443.dcm	1	0.7905
../data/osakakokusai/oosaka_pos/203.dcm	1	0.791
../data/osakakokusai/oosaka_pos/545.dcm	1	0.791
../data/osakakokusai/oosaka_pos/180.dcm	1	0.792
../data/osakakokusai/oosaka_pos/546.dcm	1	0.797
../data/osakakokusai/oosaka_pos/556.dcm	1	0.797
../data/osakakokusai/oosaka_pos/389.dcm	1	0.7974
../data/osakakokusai/oosaka_pos/501.dcm	1	0.801
../data/osakakokusai/oosaka_pos/511.dcm	1	0.8027
../data/osakakokusai/oosaka_pos/394.dcm	1	0.804
../data/osakakokusai/oosaka_pos/21.dcm	1	0.8076
../data/osakakokusai/oosaka_pos/23.dcm	1	0.8086
../data/osakakokusai/oosaka_pos/277.dcm	1	0.809
../data/osakakokusai/oosaka_pos/362.dcm	1	0.809
../data/osakakokusai/oosaka_pos/553.dcm	1	0.81
../data/osakakokusai/oosaka_pos/75.dcm	1	0.813
../data/osakakokusai/oosaka_pos/4.dcm	1	0.8135
../data/osakakokusai/oosaka_pos/6.dcm	1	0.8145
../data/osakakokusai/oosaka_pos/304.dcm	1	0.8145
../data/osakakokusai/oosaka_pos/291.dcm	1	0.8154
../data/osakakokusai/oosaka_pos/351.dcm	1	0.816
../data/osakakokusai/oosaka_pos/206.dcm	1	0.8174
../data/osakakokusai/oosaka_pos/350.dcm	1	0.8174
../data/osakakokusai/oosaka_pos/542.dcm	1	0.8174
../data/osakakokusai/oosaka_pos/325.dcm	1	0.824
../data/osakakokusai/oosaka_pos/502.dcm	1	0.825
../data/osakakokusai/oosaka_pos/387.dcm	1	0.827
../data/osakakokusai/oosaka_pos/448.dcm	1	0.8276
../data/osakakokusai/oosaka_pos/370.dcm	1	0.8286
../data/osakakokusai/oosaka_pos/253.dcm	1	0.829
../data/osakakokusai/oosaka_pos/558.dcm	1	0.829
../data/osakakokusai/oosaka_pos/8.dcm	1	0.832
../data/osakakokusai/oosaka_pos/247.dcm	1	0.8335
../data/osakakokusai/oosaka_pos/302.dcm	1	0.834
../data/osakakokusai/oosaka_pos/321.dcm	1	0.834
../data/osakakokusai/oosaka_pos/322.dcm	1	0.835
../data/osakakokusai/oosaka_pos/531.dcm	1	0.8384
../data/osakakokusai/oosaka_pos/548.dcm	1	0.8384
../data/osakakokusai/oosaka_pos/543.dcm	1	0.843
../data/osakakokusai/oosaka_pos/124.dcm	1	0.8438

../data/osakakokusai/oosaka_pos/249.dcm	1	0.845
../data/osakakokusai/oosaka_pos/555.dcm	1	0.847
../data/osakakokusai/oosaka_pos/130.dcm	1	0.849
../data/osakakokusai/oosaka_pos/450.dcm	1	0.853
../data/osakakokusai/oosaka_pos/15.dcm	1	0.8545
../data/osakakokusai/oosaka_pos/248.dcm	1	0.8555
../data/osakakokusai/oosaka_pos/315.dcm	1	0.859
../data/osakakokusai/oosaka_pos/26.dcm	1	0.86
../data/osakakokusai/oosaka_pos/299.dcm	1	0.86
../data/osakakokusai/oosaka_pos/244.dcm	1	0.861
../data/osakakokusai/oosaka_pos/371.dcm	1	0.861
../data/osakakokusai/oosaka_pos/532.dcm	1	0.863
../data/osakakokusai/oosaka_pos/174.dcm	1	0.8647
../data/osakakokusai/oosaka_pos/566.dcm	1	0.8647
../data/osakakokusai/oosaka_pos/557.dcm	1	0.8657
../data/osakakokusai/oosaka_pos/212.dcm	1	0.866
../data/osakakokusai/oosaka_pos/29.dcm	1	0.87
../data/osakakokusai/oosaka_pos/245.dcm	1	0.8706
../data/osakakokusai/oosaka_pos/551.dcm	1	0.8706
../data/osakakokusai/oosaka_pos/373.dcm	1	0.872
../data/osakakokusai/oosaka_pos/549.dcm	1	0.8726
../data/osakakokusai/oosaka_pos/554.dcm	1	0.879
../data/osakakokusai/oosaka_pos/182.dcm	1	0.883
../data/osakakokusai/oosaka_pos/379.dcm	1	0.8857
../data/osakakokusai/oosaka_pos/374.dcm	1	0.8887
../data/osakakokusai/oosaka_pos/378.dcm	1	0.8887
../data/osakakokusai/oosaka_pos/496.dcm	1	0.8965
../data/osakakokusai/oosaka_pos/183.dcm	1	0.9263
../data/osakakokusai/oosaka_pos/176.dcm	1	0.927
../data/mizushima_neg/dicom¥1.dcm	0	0.3225
../data/mizushima_neg/dicom¥101.dcm	0	0.12024
../data/mizushima_neg/dicom¥102.dcm	0	0.1705
../data/mizushima_neg/dicom¥103.dcm	0	0.5527
../data/mizushima_neg/dicom¥105.dcm	0	0.1451
../data/mizushima_neg/dicom¥106.dcm	0	0.01628
../data/mizushima_neg/dicom¥107.dcm	0	0.4688
../data/mizushima_neg/dicom¥108.dcm	0	0.2483
../data/mizushima_neg/dicom¥109.dcm	0	0.1804
../data/mizushima_neg/dicom¥11.dcm	0	0.04535
../data/mizushima_neg/dicom¥110.dcm	0	0.3674

../data/mizushima_neg/dicom¥112.dcm	0	0.0666
../data/mizushima_neg/dicom¥113.dcm	0	0.03662
../data/mizushima_neg/dicom¥114.dcm	0	0.5
../data/mizushima_neg/dicom¥115.dcm	0	0.0933
../data/mizushima_neg/dicom¥116.dcm	0	0.4387
../data/mizushima_neg/dicom¥117.dcm	0	0.355
../data/mizushima_neg/dicom¥118.dcm	0	0.1981
../data/mizushima_neg/dicom¥119.dcm	0	0.09125
../data/mizushima_neg/dicom¥12.dcm	0	0.0201
../data/mizushima_neg/dicom¥120.dcm	0	0.5273
../data/mizushima_neg/dicom¥121.dcm	0	0.183
../data/mizushima_neg/dicom¥122.dcm	0	0.00755
../data/mizushima_neg/dicom¥123.dcm	0	0.02475
../data/mizushima_neg/dicom¥124.dcm	0	0.00927
../data/mizushima_neg/dicom¥126.dcm	0	0.03622
../data/mizushima_neg/dicom¥127.dcm	0	0.2255
../data/mizushima_neg/dicom¥128.dcm	0	0.499
../data/mizushima_neg/dicom¥129.dcm	0	0.02254
../data/mizushima_neg/dicom¥132.dcm	0	0.0258
../data/mizushima_neg/dicom¥133.dcm	0	0.05203
../data/mizushima_neg/dicom¥134.dcm	0	0.01898
../data/mizushima_neg/dicom¥14.dcm	0	0.07135
../data/mizushima_neg/dicom¥141.dcm	0	0.218
../data/mizushima_neg/dicom¥142.dcm	0	0.00948
../data/mizushima_neg/dicom¥149.dcm	0	0.4736
../data/mizushima_neg/dicom¥15.dcm	0	0.006565
../data/mizushima_neg/dicom¥150.dcm	0	0.55
../data/mizushima_neg/dicom¥151.dcm	0	0.04327
../data/mizushima_neg/dicom¥152.dcm	0	0.1792
../data/mizushima_neg/dicom¥154.dcm	0	0.02876
../data/mizushima_neg/dicom¥155.dcm	0	0.01659
../data/mizushima_neg/dicom¥157.dcm	0	0.1307
../data/mizushima_neg/dicom¥158.dcm	0	0.02666
../data/mizushima_neg/dicom¥160.dcm	0	0.0334
../data/mizushima_neg/dicom¥161.dcm	0	0.0945
../data/mizushima_neg/dicom¥162.dcm	0	0.075
../data/mizushima_neg/dicom¥163.dcm	0	0.2727
../data/mizushima_neg/dicom¥164.dcm	0	0.00547
../data/mizushima_neg/dicom¥165.dcm	0	0.008064
../data/mizushima_neg/dicom¥167.dcm	0	0.0109

../data/mizushima_neg/dicom¥168.dcm	0	0.006588
../data/mizushima_neg/dicom¥17.dcm	0	0.00339
../data/mizushima_neg/dicom¥170.dcm	0	0.00378
../data/mizushima_neg/dicom¥171.dcm	0	0.008545
../data/mizushima_neg/dicom¥172.dcm	0	0.05737
../data/mizushima_neg/dicom¥173.dcm	0	0.00997
../data/mizushima_neg/dicom¥174.dcm	0	0.2546
../data/mizushima_neg/dicom¥175.dcm	0	0.2573
../data/mizushima_neg/dicom¥176.dcm	0	0.01991
../data/mizushima_neg/dicom¥177.dcm	0	0.1738
../data/mizushima_neg/dicom¥179.dcm	0	0.0355
../data/mizushima_neg/dicom¥18.dcm	0	0.002993
../data/mizushima_neg/dicom¥180.dcm	0	0.02289
../data/mizushima_neg/dicom¥181.dcm	0	0.1892
../data/mizushima_neg/dicom¥182.dcm	0	0.03705
../data/mizushima_neg/dicom¥183.dcm	0	0.1332
../data/mizushima_neg/dicom¥184.dcm	0	0.1378
../data/mizushima_neg/dicom¥185.dcm	0	0.11316
../data/mizushima_neg/dicom¥186.dcm	0	0.1545
../data/mizushima_neg/dicom¥187.dcm	0	0.06915
../data/mizushima_neg/dicom¥188.dcm	0	0.0882
../data/mizushima_neg/dicom¥19.dcm	0	0.00399
../data/mizushima_neg/dicom¥190.dcm	0	0.01628
../data/mizushima_neg/dicom¥191.dcm	0	0.2502
../data/mizushima_neg/dicom¥192.dcm	0	0.3367
../data/mizushima_neg/dicom¥193.dcm	0	0.07263
../data/mizushima_neg/dicom¥195.dcm	0	0.041
../data/mizushima_neg/dicom¥196.dcm	0	0.2028
../data/mizushima_neg/dicom¥197.dcm	0	0.10156
../data/mizushima_neg/dicom¥198.dcm	0	0.02065
../data/mizushima_neg/dicom¥199.dcm	0	0.0386
../data/mizushima_neg/dicom¥2.dcm	0	0.1616
../data/mizushima_neg/dicom¥200.dcm	0	0.06198
../data/mizushima_neg/dicom¥201.dcm	0	0.01277
../data/mizushima_neg/dicom¥203.dcm	0	0.382
../data/mizushima_neg/dicom¥204.dcm	0	0.07135
../data/mizushima_neg/dicom¥205.dcm	0	0.002958
../data/mizushima_neg/dicom¥206.dcm	0	0.003456
../data/mizushima_neg/dicom¥207.dcm	0	0.005936
../data/mizushima_neg/dicom¥209.dcm	0	0.007233

../data/mizushima_neg/dicom¥21.dcm	0	0.004135
../data/mizushima_neg/dicom¥211.dcm	0	0.008514
../data/mizushima_neg/dicom¥212.dcm	0	0.04987
../data/mizushima_neg/dicom¥213.dcm	0	0.08093
../data/mizushima_neg/dicom¥215.dcm	0	0.02342
../data/mizushima_neg/dicom¥216.dcm	0	0.1343
../data/mizushima_neg/dicom¥217.dcm	0	0.008675
../data/mizushima_neg/dicom¥218.dcm	0	0.1285
../data/mizushima_neg/dicom¥219.dcm	0	0.07544
../data/mizushima_neg/dicom¥22.dcm	0	0.002323
../data/mizushima_neg/dicom¥220.dcm	0	0.015015
../data/mizushima_neg/dicom¥221.dcm	0	0.001949
../data/mizushima_neg/dicom¥222.dcm	0	0.00982
../data/mizushima_neg/dicom¥223.dcm	0	0.0808
../data/mizushima_neg/dicom¥224.dcm	0	0.02014
../data/mizushima_neg/dicom¥226.dcm	0	0.00435
../data/mizushima_neg/dicom¥227.dcm	0	0.014114
../data/mizushima_neg/dicom¥228.dcm	0	0.003765
../data/mizushima_neg/dicom¥229.dcm	0	0.01473
../data/mizushima_neg/dicom¥231.dcm	0	0.006123
../data/mizushima_neg/dicom¥232.dcm	0	0.3965
../data/mizushima_neg/dicom¥233.dcm	0	0.3857
../data/mizushima_neg/dicom¥234.dcm	0	0.04282
../data/mizushima_neg/dicom¥235.dcm	0	0.631
../data/mizushima_neg/dicom¥236.dcm	0	0.06335
../data/mizushima_neg/dicom¥237.dcm	0	0.0857
../data/mizushima_neg/dicom¥24.dcm	0	0.1136
../data/mizushima_neg/dicom¥240.dcm	0	0.1859
../data/mizushima_neg/dicom¥241.dcm	0	0.02556
../data/mizushima_neg/dicom¥242.dcm	0	0.02855
../data/mizushima_neg/dicom¥243.dcm	0	0.07684
../data/mizushima_neg/dicom¥244.dcm	0	0.005642
../data/mizushima_neg/dicom¥245.dcm	0	0.002562
../data/mizushima_neg/dicom¥247.dcm	0	0.02414
../data/mizushima_neg/dicom¥25.dcm	0	0.0914
../data/mizushima_neg/dicom¥250.dcm	0	0.008415
../data/mizushima_neg/dicom¥251.dcm	0	0.0181
../data/mizushima_neg/dicom¥252.dcm	0	0.004868
../data/mizushima_neg/dicom¥253.dcm	0	0.04047
../data/mizushima_neg/dicom¥255.dcm	0	0.0397

../data/mizushima_neg/dicom¥256.dcm	0	0.134
../data/mizushima_neg/dicom¥257.dcm	0	0.08124
../data/mizushima_neg/dicom¥258.dcm	0	0.2274
../data/mizushima_neg/dicom¥26.dcm	0	0.03934
../data/mizushima_neg/dicom¥260.dcm	0	0.0424
../data/mizushima_neg/dicom¥261.dcm	0	0.4011
../data/mizushima_neg/dicom¥262.dcm	0	0.0613
../data/mizushima_neg/dicom¥264.dcm	0	0.2069
../data/mizushima_neg/dicom¥265.dcm	0	0.004772
../data/mizushima_neg/dicom¥266.dcm	0	0.00952
../data/mizushima_neg/dicom¥269.dcm	0	0.004593
../data/mizushima_neg/dicom¥27.dcm	0	0.03067
../data/mizushima_neg/dicom¥270.dcm	0	0.01567
../data/mizushima_neg/dicom¥271.dcm	0	0.003635
../data/mizushima_neg/dicom¥272.dcm	0	0.00755
../data/mizushima_neg/dicom¥273.dcm	0	0.005844
../data/mizushima_neg/dicom¥274.dcm	0	0.00404
../data/mizushima_neg/dicom¥275.dcm	0	0.005363
../data/mizushima_neg/dicom¥277.dcm	0	0.591
../data/mizushima_neg/dicom¥278.dcm	0	0.2118
../data/mizushima_neg/dicom¥279.dcm	0	0.01869
../data/mizushima_neg/dicom¥280.dcm	0	0.124
../data/mizushima_neg/dicom¥281.dcm	0	0.04178
../data/mizushima_neg/dicom¥282.dcm	0	0.008255
../data/mizushima_neg/dicom¥283.dcm	0	0.4
../data/mizushima_neg/dicom¥284.dcm	0	0.00986
../data/mizushima_neg/dicom¥285.dcm	0	0.007175
../data/mizushima_neg/dicom¥286.dcm	0	0.004944
../data/mizushima_neg/dicom¥287.dcm	0	0.1023
../data/mizushima_neg/dicom¥29.dcm	0	0.1105
../data/mizushima_neg/dicom¥292.dcm	0	0.06165
../data/mizushima_neg/dicom¥293.dcm	0	0.05234
../data/mizushima_neg/dicom¥295.dcm	0	0.003975
../data/mizushima_neg/dicom¥296.dcm	0	0.01238
../data/mizushima_neg/dicom¥299.dcm	0	0.263
../data/mizushima_neg/dicom¥30.dcm	0	0.0484
../data/mizushima_neg/dicom¥300.dcm	0	0.1466
../data/mizushima_neg/dicom¥301.dcm	0	0.00619
../data/mizushima_neg/dicom¥302.dcm	0	0.01257
../data/mizushima_neg/dicom¥303.dcm	0	0.01017

../data/mizushima_neg/dicom¥304.dcm	0	0.00514
../data/mizushima_neg/dicom¥305.dcm	0	0.01616
../data/mizushima_neg/dicom¥306.dcm	0	0.0051
../data/mizushima_neg/dicom¥307.dcm	0	0.04037
../data/mizushima_neg/dicom¥308.dcm	0	0.0241
../data/mizushima_neg/dicom¥31.dcm	0	0.03833
../data/mizushima_neg/dicom¥311.dcm	0	0.07465
../data/mizushima_neg/dicom¥312.dcm	0	0.1084
../data/mizushima_neg/dicom¥313.dcm	0	0.6426
../data/mizushima_neg/dicom¥314.dcm	0	0.307
../data/mizushima_neg/dicom¥315.dcm	0	0.3767
../data/mizushima_neg/dicom¥316.dcm	0	0.01009
../data/mizushima_neg/dicom¥317.dcm	0	0.012054
../data/mizushima_neg/dicom¥318.dcm	0	0.3933
../data/mizushima_neg/dicom¥32.dcm	0	0.4387
../data/mizushima_neg/dicom¥322.dcm	0	0.01116
../data/mizushima_neg/dicom¥323.dcm	0	0.008125
../data/mizushima_neg/dicom¥324.dcm	0	0.397
../data/mizushima_neg/dicom¥325.dcm	0	0.2108
../data/mizushima_neg/dicom¥326.dcm	0	0.01385
../data/mizushima_neg/dicom¥328.dcm	0	0.01359
../data/mizushima_neg/dicom¥329.dcm	0	0.01192
../data/mizushima_neg/dicom¥331.dcm	0	0.0173
../data/mizushima_neg/dicom¥333.dcm	0	0.009056
../data/mizushima_neg/dicom¥334.dcm	0	0.2418
../data/mizushima_neg/dicom¥336.dcm	0	0.02246
../data/mizushima_neg/dicom¥338.dcm	0	0.004215
../data/mizushima_neg/dicom¥339.dcm	0	0.002855
../data/mizushima_neg/dicom¥341.dcm	0	0.02686
../data/mizushima_neg/dicom¥342.dcm	0	0.009674
../data/mizushima_neg/dicom¥346.dcm	0	0.007812
../data/mizushima_neg/dicom¥347.dcm	0	0.00502
../data/mizushima_neg/dicom¥348.dcm	0	0.05814
../data/mizushima_neg/dicom¥349.dcm	0	0.0469
../data/mizushima_neg/dicom¥350.dcm	0	0.01348
../data/mizushima_neg/dicom¥351.dcm	0	0.188
../data/mizushima_neg/dicom¥353.dcm	0	0.005405
../data/mizushima_neg/dicom¥354.dcm	0	0.005
../data/mizushima_neg/dicom¥355.dcm	0	0.06915
../data/mizushima_neg/dicom¥356.dcm	0	0.1526

../data/mizushima_neg/dicom¥357.dcm	0	0.04138
../data/mizushima_neg/dicom¥358.dcm	0	0.0721
../data/mizushima_neg/dicom¥359.dcm	0	0.03607
../data/mizushima_neg/dicom¥361.dcm	0	0.02681
../data/mizushima_neg/dicom¥363.dcm	0	0.0121
../data/mizushima_neg/dicom¥364.dcm	0	0.0742
../data/mizushima_neg/dicom¥366.dcm	0	0.01888
../data/mizushima_neg/dicom¥367.dcm	0	0.01107
../data/mizushima_neg/dicom¥372.dcm	0	0.1128
../data/mizushima_neg/dicom¥373.dcm	0	0.006668
../data/mizushima_neg/dicom¥374.dcm	0	0.01906
../data/mizushima_neg/dicom¥376.dcm	0	0.04752
../data/mizushima_neg/dicom¥377.dcm	0	0.006588
../data/mizushima_neg/dicom¥379.dcm	0	0.005844
../data/mizushima_neg/dicom¥38.dcm	0	0.3452
../data/mizushima_neg/dicom¥380.dcm	0	0.113
../data/mizushima_neg/dicom¥382.dcm	0	0.006615
../data/mizushima_neg/dicom¥383.dcm	0	0.011375
../data/mizushima_neg/dicom¥385.dcm	0	0.1643
../data/mizushima_neg/dicom¥386.dcm	0	0.2203
../data/mizushima_neg/dicom¥388.dcm	0	0.539
../data/mizushima_neg/dicom¥389.dcm	0	0.4775
../data/mizushima_neg/dicom¥39.dcm	0	0.3145
../data/mizushima_neg/dicom¥390.dcm	0	0.547
../data/mizushima_neg/dicom¥391.dcm	0	0.2786
../data/mizushima_neg/dicom¥392.dcm	0	0.005665
../data/mizushima_neg/dicom¥393.dcm	0	0.02443
../data/mizushima_neg/dicom¥394.dcm	0	0.0112
../data/mizushima_neg/dicom¥395.dcm	0	0.01086
../data/mizushima_neg/dicom¥396.dcm	0	0.006668
../data/mizushima_neg/dicom¥400.dcm	0	0.00895
../data/mizushima_neg/dicom¥401.dcm	0	0.00948
../data/mizushima_neg/dicom¥402.dcm	0	0.6104
../data/mizushima_neg/dicom¥403.dcm	0	0.4282
../data/mizushima_neg/dicom¥404.dcm	0	0.234
../data/mizushima_neg/dicom¥405.dcm	0	0.001399
../data/mizushima_neg/dicom¥406.dcm	0	0.00982
../data/mizushima_neg/dicom¥407.dcm	0	0.03378
../data/mizushima_neg/dicom¥409.dcm	0	0.04068
../data/mizushima_neg/dicom¥41.dcm	0	0.01917

../data/mizushima_neg/dicom¥411.dcm	0	0.2803
../data/mizushima_neg/dicom¥412.dcm	0	0.006615
../data/mizushima_neg/dicom¥414.dcm	0	0.10596
../data/mizushima_neg/dicom¥416.dcm	0	0.008545
../data/mizushima_neg/dicom¥417.dcm	0	0.00479
../data/mizushima_neg/dicom¥419.dcm	0	0.3203
../data/mizushima_neg/dicom¥42.dcm	0	0.01196
../data/mizushima_neg/dicom¥421.dcm	0	0.01263
../data/mizushima_neg/dicom¥423.dcm	0	0.01009
../data/mizushima_neg/dicom¥424.dcm	0	0.01021
../data/mizushima_neg/dicom¥426.dcm	0	0.03824
../data/mizushima_neg/dicom¥427.dcm	0	0.01602
../data/mizushima_neg/dicom¥429.dcm	0	0.2006
../data/mizushima_neg/dicom¥430.dcm	0	0.2024
../data/mizushima_neg/dicom¥431.dcm	0	0.05997
../data/mizushima_neg/dicom¥433.dcm	0	0.02342
../data/mizushima_neg/dicom¥434.dcm	0	0.02042
../data/mizushima_neg/dicom¥435.dcm	0	0.00755
../data/mizushima_neg/dicom¥437.dcm	0	0.12067
../data/mizushima_neg/dicom¥438.dcm	0	0.08386
../data/mizushima_neg/dicom¥44.dcm	0	0.007374
../data/mizushima_neg/dicom¥440.dcm	0	0.1299
../data/mizushima_neg/dicom¥441.dcm	0	0.004383
../data/mizushima_neg/dicom¥442.dcm	0	0.00997
../data/mizushima_neg/dicom¥444.dcm	0	0.04434
../data/mizushima_neg/dicom¥446.dcm	0	0.01456
../data/mizushima_neg/dicom¥448.dcm	0	0.00365
../data/mizushima_neg/dicom¥449.dcm	0	0.0121
../data/mizushima_neg/dicom¥45.dcm	0	0.0345
../data/mizushima_neg/dicom¥450.dcm	0	0.05878
../data/mizushima_neg/dicom¥451.dcm	0	0.00516
../data/mizushima_neg/dicom¥452.dcm	0	0.01628
../data/mizushima_neg/dicom¥453.dcm	0	0.2118
../data/mizushima_neg/dicom¥454.dcm	0	0.0332
../data/mizushima_neg/dicom¥455.dcm	0	0.4824
../data/mizushima_neg/dicom¥456.dcm	0	0.006798
../data/mizushima_neg/dicom¥458.dcm	0	0.01718
../data/mizushima_neg/dicom¥459.dcm	0	0.002823
../data/mizushima_neg/dicom¥46.dcm	0	0.0899
../data/mizushima_neg/dicom¥460.dcm	0	0.015015

../data/mizushima_neg/dicom¥461.dcm	0	0.02533
../data/mizushima_neg/dicom¥462.dcm	0	0.2021
../data/mizushima_neg/dicom¥464.dcm	0	0.0156
../data/mizushima_neg/dicom¥465.dcm	0	0.00479
../data/mizushima_neg/dicom¥467.dcm	0	0.00885
../data/mizushima_neg/dicom¥468.dcm	0	0.009415
../data/mizushima_neg/dicom¥47.dcm	0	0.01297
../data/mizushima_neg/dicom¥470.dcm	0	0.00835
../data/mizushima_neg/dicom¥471.dcm	0	0.00454
../data/mizushima_neg/dicom¥478.dcm	0	0.0184
../data/mizushima_neg/dicom¥479.dcm	0	0.07184
../data/mizushima_neg/dicom¥480.dcm	0	0.0876
../data/mizushima_neg/dicom¥481.dcm	0	0.0367
../data/mizushima_neg/dicom¥484.dcm	0	0.00428
../data/mizushima_neg/dicom¥485.dcm	0	0.11163
../data/mizushima_neg/dicom¥487.dcm	0	0.002531
../data/mizushima_neg/dicom¥488.dcm	0	0.02438
../data/mizushima_neg/dicom¥489.dcm	0	0.01495
../data/mizushima_neg/dicom¥491.dcm	0	0.07806
../data/mizushima_neg/dicom¥492.dcm	0	0.01178
../data/mizushima_neg/dicom¥493.dcm	0	0.006218
../data/mizushima_neg/dicom¥494.dcm	0	0.05542
../data/mizushima_neg/dicom¥499.dcm	0	0.0883
../data/mizushima_neg/dicom¥5.dcm	0	0.569
../data/mizushima_neg/dicom¥500.dcm	0	0.05167
../data/mizushima_neg/dicom¥502.dcm	0	0.00502
../data/mizushima_neg/dicom¥504.dcm	0	0.09534
../data/mizushima_neg/dicom¥505.dcm	0	0.001839
../data/mizushima_neg/dicom¥507.dcm	0	0.008644
../data/mizushima_neg/dicom¥508.dcm	0	0.005554
../data/mizushima_neg/dicom¥51.dcm	0	0.002216
../data/mizushima_neg/dicom¥510.dcm	0	0.03198
../data/mizushima_neg/dicom¥511.dcm	0	0.04303
../data/mizushima_neg/dicom¥512.dcm	0	0.01791
../data/mizushima_neg/dicom¥513.dcm	0	0.01045
../data/mizushima_neg/dicom¥514.dcm	0	0.02948
../data/mizushima_neg/dicom¥515.dcm	0	0.004906
../data/mizushima_neg/dicom¥516.dcm	0	0.0189
../data/mizushima_neg/dicom¥518.dcm	0	0.1252
../data/mizushima_neg/dicom¥519.dcm	0	0.0505

../data/mizushima_neg/dicom¥52.dcm	0	0.10266
../data/mizushima_neg/dicom¥521.dcm	0	0.02666
../data/mizushima_neg/dicom¥522.dcm	0	0.00726
../data/mizushima_neg/dicom¥524.dcm	0	0.02211
../data/mizushima_neg/dicom¥525.dcm	0	0.01602
../data/mizushima_neg/dicom¥527.dcm	0	0.004944
../data/mizushima_neg/dicom¥528.dcm	0	0.01057
../data/mizushima_neg/dicom¥53.dcm	0	0.4197
../data/mizushima_neg/dicom¥531.dcm	0	0.04147
../data/mizushima_neg/dicom¥532.dcm	0	0.08417
../data/mizushima_neg/dicom¥537.dcm	0	0.001346
../data/mizushima_neg/dicom¥538.dcm	0	0.004906
../data/mizushima_neg/dicom¥539.dcm	0	0.1366
../data/mizushima_neg/dicom¥54.dcm	0	0.00881
../data/mizushima_neg/dicom¥540.dcm	0	0.01956
../data/mizushima_neg/dicom¥541.dcm	0	0.0775
../data/mizushima_neg/dicom¥542.dcm	0	0.007576
../data/mizushima_neg/dicom¥543.dcm	0	0.0188
../data/mizushima_neg/dicom¥544.dcm	0	0.011505
../data/mizushima_neg/dicom¥546.dcm	0	0.1807
../data/mizushima_neg/dicom¥547.dcm	0	0.0173
../data/mizushima_neg/dicom¥549.dcm	0	0.02072
../data/mizushima_neg/dicom¥55.dcm	0	0.591
../data/mizushima_neg/dicom¥550.dcm	0	0.01243
../data/mizushima_neg/dicom¥551.dcm	0	0.002684
../data/mizushima_neg/dicom¥552.dcm	0	0.02211
../data/mizushima_neg/dicom¥554.dcm	0	0.001649
../data/mizushima_neg/dicom¥555.dcm	0	0.05145
../data/mizushima_neg/dicom¥556.dcm	0	0.007607
../data/mizushima_neg/dicom¥558.dcm	0	0.03174
../data/mizushima_neg/dicom¥559.dcm	0	0.01263
../data/mizushima_neg/dicom¥56.dcm	0	0.01646
../data/mizushima_neg/dicom¥564.dcm	0	0.01484
../data/mizushima_neg/dicom¥565.dcm	0	0.065
../data/mizushima_neg/dicom¥566.dcm	0	0.002823
../data/mizushima_neg/dicom¥567.dcm	0	0.0021
../data/mizushima_neg/dicom¥569.dcm	0	0.0241
../data/mizushima_neg/dicom¥570.dcm	0	0.014114
../data/mizushima_neg/dicom¥571.dcm	0	0.02562
../data/mizushima_neg/dicom¥572.dcm	0	0.05603

../data/mizushima_neg/dicom¥574.dcm	0	0.01363
../data/mizushima_neg/dicom¥575.dcm	0	0.008255
../data/mizushima_neg/dicom¥577.dcm	0	0.002768
../data/mizushima_neg/dicom¥578.dcm	0	0.01133
../data/mizushima_neg/dicom¥58.dcm	0	0.04587
../data/mizushima_neg/dicom¥580.dcm	0	0.007725
../data/mizushima_neg/dicom¥581.dcm	0	0.04248
../data/mizushima_neg/dicom¥583.dcm	0	0.27
../data/mizushima_neg/dicom¥584.dcm	0	0.0979
../data/mizushima_neg/dicom¥585.dcm	0	0.1925
../data/mizushima_neg/dicom¥586.dcm	0	0.00368
../data/mizushima_neg/dicom¥588.dcm	0	0.412
../data/mizushima_neg/dicom¥59.dcm	0	0.002913
../data/mizushima_neg/dicom¥590.dcm	0	0.1901
../data/mizushima_neg/dicom¥591.dcm	0	0.0693
../data/mizushima_neg/dicom¥592.dcm	0	0.0548
../data/mizushima_neg/dicom¥594.dcm	0	0.616
../data/mizushima_neg/dicom¥595.dcm	0	0.00701
../data/mizushima_neg/dicom¥596.dcm	0	0.155
../data/mizushima_neg/dicom¥597.dcm	0	0.01041
../data/mizushima_neg/dicom¥598.dcm	0	0.02203
../data/mizushima_neg/dicom¥599.dcm	0	0.01439
../data/mizushima_neg/dicom¥6.dcm	0	0.00982
../data/mizushima_neg/dicom¥60.dcm	0	0.007404
../data/mizushima_neg/dicom¥601.dcm	0	0.381
../data/mizushima_neg/dicom¥603.dcm	0	0.0801
../data/mizushima_neg/dicom¥604.dcm	0	0.02187
../data/mizushima_neg/dicom¥605.dcm	0	0.2024
../data/mizushima_neg/dicom¥606.dcm	0	0.007786
../data/mizushima_neg/dicom¥607.dcm	0	0.03705
../data/mizushima_neg/dicom¥608.dcm	0	0.02744
../data/mizushima_neg/dicom¥609.dcm	0	0.00468
../data/mizushima_neg/dicom¥610.dcm	0	0.00358
../data/mizushima_neg/dicom¥611.dcm	0	0.006615
../data/mizushima_neg/dicom¥613.dcm	0	0.02298
../data/mizushima_neg/dicom¥614.dcm	0	0.00423
../data/mizushima_neg/dicom¥616.dcm	0	0.658
../data/mizushima_neg/dicom¥617.dcm	0	0.4495
../data/mizushima_neg/dicom¥619.dcm	0	0.01938
../data/mizushima_neg/dicom¥62.dcm	0	0.0145

../data/mizushima_neg/dicom¥620.dcm	0	0.011505
../data/mizushima_neg/dicom¥622.dcm	0	0.0205
../data/mizushima_neg/dicom¥623.dcm	0	0.1536
../data/mizushima_neg/dicom¥625.dcm	0	0.0457
../data/mizushima_neg/dicom¥626.dcm	0	0.002747
../data/mizushima_neg/dicom¥628.dcm	0	0.01257
../data/mizushima_neg/dicom¥629.dcm	0	0.00461
../data/mizushima_neg/dicom¥63.dcm	0	0.2903
../data/mizushima_neg/dicom¥631.dcm	0	0.05542
../data/mizushima_neg/dicom¥632.dcm	0	0.003593
../data/mizushima_neg/dicom¥636.dcm	0	0.0149
../data/mizushima_neg/dicom¥637.dcm	0	0.03062
../data/mizushima_neg/dicom¥640.dcm	0	0.014114
../data/mizushima_neg/dicom¥641.dcm	0	0.007694
../data/mizushima_neg/dicom¥642.dcm	0	0.00514
../data/mizushima_neg/dicom¥643.dcm	0	0.02701
../data/mizushima_neg/dicom¥645.dcm	0	0.0041
../data/mizushima_neg/dicom¥646.dcm	0	0.1526
../data/mizushima_neg/dicom¥652.dcm	0	0.02988
../data/mizushima_neg/dicom¥653.dcm	0	0.01578
../data/mizushima_neg/dicom¥654.dcm	0	0.0379
../data/mizushima_neg/dicom¥656.dcm	0	0.006462
../data/mizushima_neg/dicom¥658.dcm	0	0.02827
../data/mizushima_neg/dicom¥660.dcm	0	0.384
../data/mizushima_neg/dicom¥662.dcm	0	0.2998
../data/mizushima_neg/dicom¥663.dcm	0	0.4543
../data/mizushima_neg/dicom¥664.dcm	0	0.05728
../data/mizushima_neg/dicom¥665.dcm	0	0.1249
../data/mizushima_neg/dicom¥666.dcm	0	0.222
../data/mizushima_neg/dicom¥667.dcm	0	0.01129
../data/mizushima_neg/dicom¥668.dcm	0	0.2893
../data/mizushima_neg/dicom¥669.dcm	0	0.03348
../data/mizushima_neg/dicom¥67.dcm	0	0.263
../data/mizushima_neg/dicom¥670.dcm	0	0.00502
../data/mizushima_neg/dicom¥673.dcm	0	0.05072
../data/mizushima_neg/dicom¥674.dcm	0	0.011505
../data/mizushima_neg/dicom¥675.dcm	0	0.4795
../data/mizushima_neg/dicom¥676.dcm	0	0.005913
../data/mizushima_neg/dicom¥677.dcm	0	0.02089
../data/mizushima_neg/dicom¥68.dcm	0	0.003593

../data/mizushima_neg/dicom¥7.dcm	0	0.003944
../data/mizushima_neg/dicom¥71.dcm	0	0.1744
../data/mizushima_neg/dicom¥72.dcm	0	0.0532
../data/mizushima_neg/dicom¥73.dcm	0	0.005688
../data/mizushima_neg/dicom¥75.dcm	0	0.0542
../data/mizushima_neg/dicom¥76.dcm	0	0.2512
../data/mizushima_neg/dicom¥77.dcm	0	0.04733
../data/mizushima_neg/dicom¥78.dcm	0	0.009125
../data/mizushima_neg/dicom¥8.dcm	0	0.002653
../data/mizushima_neg/dicom¥80.dcm	0	0.01495
../data/mizushima_neg/dicom¥81.dcm	0	0.04178
../data/mizushima_neg/dicom¥82.dcm	0	0.002632
../data/mizushima_neg/dicom¥83.dcm	0	0.035
../data/mizushima_neg/dicom¥84.dcm	0	0.03583
../data/mizushima_neg/dicom¥85.dcm	0	0.008644
../data/mizushima_neg/dicom¥86.dcm	0	0.02586
../data/mizushima_neg/dicom¥88.dcm	0	0.011826
../data/mizushima_neg/dicom¥9.dcm	0	0.0292
../data/mizushima_neg/dicom¥90.dcm	0	0.011375
../data/mizushima_neg/dicom¥95.dcm	0	0.03137
../data/mizushima_neg/dicom¥96.dcm	0	0.02434
../data/mizushima_neg/dicom¥97.dcm	0	0.06854
../data/mizushima_neg/dicom¥98.dcm	0	0.01549
../data/mizushima_neg/dicom¥99.dcm	0	0.1103
