# Supplementary material for: Utility of Same-Modality, Cross-Domain Transfer Learning for Malignant Bone Tumor Detection on Radiographs: A Multi-Faceted Performance Comparison with a Scratch-Trained Model
Source: Cancers (Basel). 2025 Sep 27;17(19):3144. doi: 10.3390/cancers17193144 (PMC12523960; doi:10.3390/cancers17193144)
Supplement: Supplementary file 1 [file cancers-17-03144-s001.zip › Table S2. YOLO-TL.pdf]

filename	label	score
../data/osakakokusai/oosaka_pos/517.dcm	1	0.000646
../data/osakakokusai/oosaka_pos/239.dcm	1	0.000682
../data/osakakokusai/oosaka_pos/293.dcm	1	0.000764
../data/osakakokusai/oosaka_pos/445.dcm	1	0.000922
../data/osakakokusai/oosaka_pos/480.dcm	1	0.001221
../data/osakakokusai/oosaka_pos/241.dcm	1	0.001289
../data/osakakokusai/oosaka_pos/518.dcm	1	0.001513
../data/osakakokusai/oosaka_pos/237.dcm	1	0.0021
../data/osakakokusai/oosaka_pos/447.dcm	1	0.002314
../data/osakakokusai/oosaka_pos/190.dcm	1	0.004963
../data/osakakokusai/oosaka_pos/145.dcm	1	0.00526
../data/osakakokusai/oosaka_pos/463.dcm	1	0.00596
../data/osakakokusai/oosaka_pos/230.dcm	1	0.006218
../data/osakakokusai/oosaka_pos/459.dcm	1	0.008064
../data/osakakokusai/oosaka_pos/240.dcm	1	0.00861
../data/osakakokusai/oosaka_pos/73.dcm	1	0.009636
../data/osakakokusai/oosaka_pos/150.dcm	1	0.01374
../data/osakakokusai/oosaka_pos/148.dcm	1	0.01799
../data/osakakokusai/oosaka_pos/470.dcm	1	0.02124
../data/osakakokusai/oosaka_pos/146.dcm	1	0.02333
../data/osakakokusai/oosaka_pos/457.dcm	1	0.02791
../data/osakakokusai/oosaka_pos/134.dcm	1	0.02844
../data/osakakokusai/oosaka_pos/472.dcm	1	0.02876
../data/osakakokusai/oosaka_pos/136.dcm	1	0.0371
../data/osakakokusai/oosaka_pos/135.dcm	1	0.0372
../data/osakakokusai/oosaka_pos/152.dcm	1	0.0386
../data/osakakokusai/oosaka_pos/341.dcm	1	0.04132
../data/osakakokusai/oosaka_pos/426.dcm	1	0.04596
../data/osakakokusai/oosaka_pos/471.dcm	1	0.04996
../data/osakakokusai/oosaka_pos/483.dcm	1	0.05154
../data/osakakokusai/oosaka_pos/236.dcm	1	0.05212
../data/osakakokusai/oosaka_pos/469.dcm	1	0.05685
../data/osakakokusai/oosaka_pos/89.dcm	1	0.05844
../data/osakakokusai/oosaka_pos/460.dcm	1	0.05933
../data/osakakokusai/oosaka_pos/160.dcm	1	0.05975
../data/osakakokusai/oosaka_pos/147.dcm	1	0.0613
../data/osakakokusai/oosaka_pos/238.dcm	1	0.06165
../data/osakakokusai/oosaka_pos/58.dcm	1	0.0628
../data/osakakokusai/oosaka_pos/547.dcm	1	0.0656

../data/osakakokusai/oosaka_pos/425.dcm	1	0.0724
../data/osakakokusai/oosaka_pos/462.dcm	1	0.0974
../data/osakakokusai/oosaka_pos/297.dcm	1	0.0977
../data/osakakokusai/oosaka_pos/158.dcm	1	0.0991
../data/osakakokusai/oosaka_pos/349.dcm	1	0.1021
../data/osakakokusai/oosaka_pos/476.dcm	1	0.1158
../data/osakakokusai/oosaka_pos/144.dcm	1	0.12
../data/osakakokusai/oosaka_pos/423.dcm	1	0.12067
../data/osakakokusai/oosaka_pos/504.dcm	1	0.1272
../data/osakakokusai/oosaka_pos/458.dcm	1	0.128
../data/osakakokusai/oosaka_pos/93.dcm	1	0.1437
../data/osakakokusai/oosaka_pos/565.dcm	1	0.1439
../data/osakakokusai/oosaka_pos/475.dcm	1	0.1681
../data/osakakokusai/oosaka_pos/388.dcm	1	0.1896
../data/osakakokusai/oosaka_pos/71.dcm	1	0.2063
../data/osakakokusai/oosaka_pos/456.dcm	1	0.2075
../data/osakakokusai/oosaka_pos/90.dcm	1	0.2173
../data/osakakokusai/oosaka_pos/229.dcm	1	0.2437
../data/osakakokusai/oosaka_pos/422.dcm	1	0.2542
../data/osakakokusai/oosaka_pos/559.dcm	1	0.2554
../data/osakakokusai/oosaka_pos/346.dcm	1	0.2737
../data/osakakokusai/oosaka_pos/32.dcm	1	0.2947
../data/osakakokusai/oosaka_pos/259.dcm	1	0.311
../data/osakakokusai/oosaka_pos/455.dcm	1	0.3496
../data/osakakokusai/oosaka_pos/86.dcm	1	0.3877
../data/osakakokusai/oosaka_pos/101.dcm	1	0.3943
../data/osakakokusai/oosaka_pos/64.dcm	1	0.3984
../data/osakakokusai/oosaka_pos/562.dcm	1	0.402
../data/osakakokusai/oosaka_pos/477.dcm	1	0.4092
../data/osakakokusai/oosaka_pos/474.dcm	1	0.4111
../data/osakakokusai/oosaka_pos/108.dcm	1	0.4143
../data/osakakokusai/oosaka_pos/137.dcm	1	0.4177
../data/osakakokusai/oosaka_pos/95.dcm	1	0.4187
../data/osakakokusai/oosaka_pos/546.dcm	1	0.4216
../data/osakakokusai/oosaka_pos/105.dcm	1	0.4253
../data/osakakokusai/oosaka_pos/85.dcm	1	0.4263
../data/osakakokusai/oosaka_pos/88.dcm	1	0.4282
../data/osakakokusai/oosaka_pos/428.dcm	1	0.433
../data/osakakokusai/oosaka_pos/282.dcm	1	0.4368
../data/osakakokusai/oosaka_pos/79.dcm	1	0.4524

../data/osakakokusai/oosaka_pos/91.dcm	1	0.461
../data/osakakokusai/oosaka_pos/143.dcm	1	0.4639
../data/osakakokusai/oosaka_pos/339.dcm	1	0.4688
../data/osakakokusai/oosaka_pos/6.dcm	1	0.4707
../data/osakakokusai/oosaka_pos/406.dcm	1	0.4746
../data/osakakokusai/oosaka_pos/566.dcm	1	0.4922
../data/osakakokusai/oosaka_pos/70.dcm	1	0.507
../data/osakakokusai/oosaka_pos/72.dcm	1	0.5156
../data/osakakokusai/oosaka_pos/352.dcm	1	0.5264
../data/osakakokusai/oosaka_pos/94.dcm	1	0.536
../data/osakakokusai/oosaka_pos/118.dcm	1	0.537
../data/osakakokusai/oosaka_pos/11.dcm	1	0.549
../data/osakakokusai/oosaka_pos/286.dcm	1	0.565
../data/osakakokusai/oosaka_pos/87.dcm	1	0.567
../data/osakakokusai/oosaka_pos/540.dcm	1	0.568
../data/osakakokusai/oosaka_pos/60.dcm	1	0.57
../data/osakakokusai/oosaka_pos/317.dcm	1	0.571
../data/osakakokusai/oosaka_pos/325.dcm	1	0.5747
../data/osakakokusai/oosaka_pos/473.dcm	1	0.5796
../data/osakakokusai/oosaka_pos/8.dcm	1	0.592
../data/osakakokusai/oosaka_pos/75.dcm	1	0.592
../data/osakakokusai/oosaka_pos/52.dcm	1	0.6187
../data/osakakokusai/oosaka_pos/482.dcm	1	0.625
../data/osakakokusai/oosaka_pos/421.dcm	1	0.626
../data/osakakokusai/oosaka_pos/323.dcm	1	0.629
../data/osakakokusai/oosaka_pos/251.dcm	1	0.631
../data/osakakokusai/oosaka_pos/394.dcm	1	0.6323
../data/osakakokusai/oosaka_pos/289.dcm	1	0.6353
../data/osakakokusai/oosaka_pos/417.dcm	1	0.6436
../data/osakakokusai/oosaka_pos/44.dcm	1	0.647
../data/osakakokusai/oosaka_pos/413.dcm	1	0.655
../data/osakakokusai/oosaka_pos/295.dcm	1	0.6567
../data/osakakokusai/oosaka_pos/441.dcm	1	0.6567
../data/osakakokusai/oosaka_pos/48.dcm	1	0.658
../data/osakakokusai/oosaka_pos/194.dcm	1	0.6655
../data/osakakokusai/oosaka_pos/329.dcm	1	0.6655
../data/osakakokusai/oosaka_pos/285.dcm	1	0.668
../data/osakakokusai/oosaka_pos/178.dcm	1	0.68
../data/osakakokusai/oosaka_pos/499.dcm	1	0.6807
../data/osakakokusai/oosaka_pos/5.dcm	1	0.6826

../data/osakakokusai/oosaka_pos/46.dcm	1	0.6885
../data/osakakokusai/oosaka_pos/430.dcm	1	0.6895
../data/osakakokusai/oosaka_pos/442.dcm	1	0.6895
../data/osakakokusai/oosaka_pos/396.dcm	1	0.705
../data/osakakokusai/oosaka_pos/291.dcm	1	0.708
../data/osakakokusai/oosaka_pos/248.dcm	1	0.71
../data/osakakokusai/oosaka_pos/281.dcm	1	0.71
../data/osakakokusai/oosaka_pos/449.dcm	1	0.713
../data/osakakokusai/oosaka_pos/313.dcm	1	0.717
../data/osakakokusai/oosaka_pos/186.dcm	1	0.7217
../data/osakakokusai/oosaka_pos/561.dcm	1	0.7285
../data/osakakokusai/oosaka_pos/464.dcm	1	0.7305
../data/osakakokusai/oosaka_pos/3.dcm	1	0.731
../data/osakakokusai/oosaka_pos/327.dcm	1	0.7373
../data/osakakokusai/oosaka_pos/130.dcm	1	0.738
../data/osakakokusai/oosaka_pos/502.dcm	1	0.738
../data/osakakokusai/oosaka_pos/131.dcm	1	0.7407
../data/osakakokusai/oosaka_pos/4.dcm	1	0.742
../data/osakakokusai/oosaka_pos/392.dcm	1	0.744
../data/osakakokusai/oosaka_pos/409.dcm	1	0.7485
../data/osakakokusai/oosaka_pos/120.dcm	1	0.75
../data/osakakokusai/oosaka_pos/199.dcm	1	0.752
../data/osakakokusai/oosaka_pos/322.dcm	1	0.753
../data/osakakokusai/oosaka_pos/50.dcm	1	0.757
../data/osakakokusai/oosaka_pos/443.dcm	1	0.758
../data/osakakokusai/oosaka_pos/359.dcm	1	0.7583
../data/osakakokusai/oosaka_pos/563.dcm	1	0.76
../data/osakakokusai/oosaka_pos/253.dcm	1	0.7607
../data/osakakokusai/oosaka_pos/512.dcm	1	0.7637
../data/osakakokusai/oosaka_pos/246.dcm	1	0.766
../data/osakakokusai/oosaka_pos/387.dcm	1	0.7676
../data/osakakokusai/oosaka_pos/451.dcm	1	0.7705
../data/osakakokusai/oosaka_pos/122.dcm	1	0.772
../data/osakakokusai/oosaka_pos/302.dcm	1	0.7725
../data/osakakokusai/oosaka_pos/244.dcm	1	0.774
../data/osakakokusai/oosaka_pos/543.dcm	1	0.7744
../data/osakakokusai/oosaka_pos/173.dcm	1	0.779
../data/osakakokusai/oosaka_pos/398.dcm	1	0.779
../data/osakakokusai/oosaka_pos/37.dcm	1	0.786
../data/osakakokusai/oosaka_pos/415.dcm	1	0.79

../data/osakakokusai/oosaka_pos/277.dcm	1	0.791
../data/osakakokusai/oosaka_pos/74.dcm	1	0.793
../data/osakakokusai/oosaka_pos/390.dcm	1	0.7964
../data/osakakokusai/oosaka_pos/13.dcm	1	0.8027
../data/osakakokusai/oosaka_pos/9.dcm	1	0.803
../data/osakakokusai/oosaka_pos/303.dcm	1	0.806
../data/osakakokusai/oosaka_pos/62.dcm	1	0.8066
../data/osakakokusai/oosaka_pos/36.dcm	1	0.8076
../data/osakakokusai/oosaka_pos/271.dcm	1	0.8076
../data/osakakokusai/oosaka_pos/360.dcm	1	0.8105
../data/osakakokusai/oosaka_pos/544.dcm	1	0.8125
../data/osakakokusai/oosaka_pos/319.dcm	1	0.814
../data/osakakokusai/oosaka_pos/539.dcm	1	0.8145
../data/osakakokusai/oosaka_pos/424.dcm	1	0.8154
../data/osakakokusai/oosaka_pos/107.dcm	1	0.816
../data/osakakokusai/oosaka_pos/188.dcm	1	0.8164
../data/osakakokusai/oosaka_pos/363.dcm	1	0.8164
../data/osakakokusai/oosaka_pos/362.dcm	1	0.817
../data/osakakokusai/oosaka_pos/15.dcm	1	0.8174
../data/osakakokusai/oosaka_pos/304.dcm	1	0.82
../data/osakakokusai/oosaka_pos/310.dcm	1	0.8203
../data/osakakokusai/oosaka_pos/103.dcm	1	0.821
../data/osakakokusai/oosaka_pos/207.dcm	1	0.8237
../data/osakakokusai/oosaka_pos/23.dcm	1	0.8276
../data/osakakokusai/oosaka_pos/247.dcm	1	0.8276
../data/osakakokusai/oosaka_pos/299.dcm	1	0.8296
../data/osakakokusai/oosaka_pos/528.dcm	1	0.83
../data/osakakokusai/oosaka_pos/531.dcm	1	0.832
../data/osakakokusai/oosaka_pos/354.dcm	1	0.834
../data/osakakokusai/oosaka_pos/560.dcm	1	0.8345
../data/osakakokusai/oosaka_pos/77.dcm	1	0.8374
../data/osakakokusai/oosaka_pos/211.dcm	1	0.843
../data/osakakokusai/oosaka_pos/416.dcm	1	0.848
../data/osakakokusai/oosaka_pos/43.dcm	1	0.849
../data/osakakokusai/oosaka_pos/350.dcm	1	0.8525
../data/osakakokusai/oosaka_pos/537.dcm	1	0.8535
../data/osakakokusai/oosaka_pos/212.dcm	1	0.8545
../data/osakakokusai/oosaka_pos/192.dcm	1	0.8564
../data/osakakokusai/oosaka_pos/400.dcm	1	0.8564
../data/osakakokusai/oosaka_pos/301.dcm	1	0.8584

../data/osakakokusai/oosaka_pos/361.dcm	1	0.861
../data/osakakokusai/oosaka_pos/555.dcm	1	0.864
../data/osakakokusai/oosaka_pos/535.dcm	1	0.8647
../data/osakakokusai/oosaka_pos/45.dcm	1	0.868
../data/osakakokusai/oosaka_pos/99.dcm	1	0.868
../data/osakakokusai/oosaka_pos/365.dcm	1	0.8687
../data/osakakokusai/oosaka_pos/7.dcm	1	0.869
../data/osakakokusai/oosaka_pos/180.dcm	1	0.87
../data/osakakokusai/oosaka_pos/448.dcm	1	0.8706
../data/osakakokusai/oosaka_pos/174.dcm	1	0.871
../data/osakakokusai/oosaka_pos/345.dcm	1	0.8716
../data/osakakokusai/oosaka_pos/545.dcm	1	0.872
../data/osakakokusai/oosaka_pos/450.dcm	1	0.8726
../data/osakakokusai/oosaka_pos/250.dcm	1	0.873
../data/osakakokusai/oosaka_pos/206.dcm	1	0.8745
../data/osakakokusai/oosaka_pos/49.dcm	1	0.875
../data/osakakokusai/oosaka_pos/542.dcm	1	0.8755
../data/osakakokusai/oosaka_pos/395.dcm	1	0.8765
../data/osakakokusai/oosaka_pos/47.dcm	1	0.8784
../data/osakakokusai/oosaka_pos/533.dcm	1	0.8784
../data/osakakokusai/oosaka_pos/553.dcm	1	0.879
../data/osakakokusai/oosaka_pos/391.dcm	1	0.8804
../data/osakakokusai/oosaka_pos/21.dcm	1	0.881
../data/osakakokusai/oosaka_pos/309.dcm	1	0.8823
../data/osakakokusai/oosaka_pos/133.dcm	1	0.8843
../data/osakakokusai/oosaka_pos/201.dcm	1	0.885
../data/osakakokusai/oosaka_pos/389.dcm	1	0.8906
../data/osakakokusai/oosaka_pos/526.dcm	1	0.891
../data/osakakokusai/oosaka_pos/564.dcm	1	0.8916
../data/osakakokusai/oosaka_pos/348.dcm	1	0.893
../data/osakakokusai/oosaka_pos/503.dcm	1	0.895
../data/osakakokusai/oosaka_pos/370.dcm	1	0.8955
../data/osakakokusai/oosaka_pos/393.dcm	1	0.8955
../data/osakakokusai/oosaka_pos/182.dcm	1	0.8975
../data/osakakokusai/oosaka_pos/556.dcm	1	0.8984
../data/osakakokusai/oosaka_pos/558.dcm	1	0.899
../data/osakakokusai/oosaka_pos/315.dcm	1	0.9014
../data/osakakokusai/oosaka_pos/124.dcm	1	0.9033
../data/osakakokusai/oosaka_pos/209.dcm	1	0.9033
../data/osakakokusai/oosaka_pos/378.dcm	1	0.9033

../data/osakakokusai/oosaka_pos/351.dcm	1	0.9043
../data/osakakokusai/oosaka_pos/203.dcm	1	0.905
../data/osakakokusai/oosaka_pos/249.dcm	1	0.906
../data/osakakokusai/oosaka_pos/536.dcm	1	0.906
../data/osakakokusai/oosaka_pos/496.dcm	1	0.907
../data/osakakokusai/oosaka_pos/549.dcm	1	0.907
../data/osakakokusai/oosaka_pos/557.dcm	1	0.907
../data/osakakokusai/oosaka_pos/511.dcm	1	0.9077
../data/osakakokusai/oosaka_pos/369.dcm	1	0.9087
../data/osakakokusai/oosaka_pos/551.dcm	1	0.9087
../data/osakakokusai/oosaka_pos/205.dcm	1	0.9136
../data/osakakokusai/oosaka_pos/321.dcm	1	0.9136
../data/osakakokusai/oosaka_pos/532.dcm	1	0.9136
../data/osakakokusai/oosaka_pos/78.dcm	1	0.914
../data/osakakokusai/oosaka_pos/196.dcm	1	0.914
../data/osakakokusai/oosaka_pos/554.dcm	1	0.916
../data/osakakokusai/oosaka_pos/245.dcm	1	0.9194
../data/osakakokusai/oosaka_pos/26.dcm	1	0.9204
../data/osakakokusai/oosaka_pos/501.dcm	1	0.923
../data/osakakokusai/oosaka_pos/538.dcm	1	0.9253
../data/osakakokusai/oosaka_pos/379.dcm	1	0.926
../data/osakakokusai/oosaka_pos/372.dcm	1	0.928
../data/osakakokusai/oosaka_pos/29.dcm	1	0.9297
../data/osakakokusai/oosaka_pos/176.dcm	1	0.9307
../data/osakakokusai/oosaka_pos/548.dcm	1	0.933
../data/osakakokusai/oosaka_pos/183.dcm	1	0.935
../data/osakakokusai/oosaka_pos/373.dcm	1	0.9395
../data/osakakokusai/oosaka_pos/371.dcm	1	0.95
../data/osakakokusai/oosaka_pos/374.dcm	1	0.9546
../data/mizushima_neg/dicom¥1.dcm	0	0.12146
../data/mizushima_neg/dicom¥101.dcm	0	0.02089
../data/mizushima_neg/dicom¥102.dcm	0	0.001427
../data/mizushima_neg/dicom¥103.dcm	0	0.001099
../data/mizushima_neg/dicom¥105.dcm	0	0.002747
../data/mizushima_neg/dicom¥106.dcm	0	0.001597
../data/mizushima_neg/dicom¥107.dcm	0	0.06866
../data/mizushima_neg/dicom¥108.dcm	0	0.1643
../data/mizushima_neg/dicom¥109.dcm	0	0.02162
../data/mizushima_neg/dicom¥11.dcm	0	0.00384
../data/mizushima_neg/dicom¥110.dcm	0	0.12024

../data/mizushima_neg/dicom¥112.dcm	0	0.00978
../data/mizushima_neg/dicom¥113.dcm	0	0.00399
../data/mizushima_neg/dicom¥114.dcm	0	0.1857
../data/mizushima_neg/dicom¥115.dcm	0	0.014336
../data/mizushima_neg/dicom¥116.dcm	0	0.2065
../data/mizushima_neg/dicom¥117.dcm	0	0.008575
../data/mizushima_neg/dicom¥118.dcm	0	0.00619
../data/mizushima_neg/dicom¥119.dcm	0	0.03555
../data/mizushima_neg/dicom¥12.dcm	0	0.002243
../data/mizushima_neg/dicom¥120.dcm	0	0.025
../data/mizushima_neg/dicom¥121.dcm	0	0.08154
../data/mizushima_neg/dicom¥122.dcm	0	0.002684
../data/mizushima_neg/dicom¥123.dcm	0	0.002226
../data/mizushima_neg/dicom¥124.dcm	0	0.006958
../data/mizushima_neg/dicom¥126.dcm	0	0.000477
../data/mizushima_neg/dicom¥127.dcm	0	0.003885
../data/mizushima_neg/dicom¥128.dcm	0	0.04742
../data/mizushima_neg/dicom¥129.dcm	0	0.02284
../data/mizushima_neg/dicom¥132.dcm	0	0.00075
../data/mizushima_neg/dicom¥133.dcm	0	0.000544
../data/mizushima_neg/dicom¥134.dcm	0	0.003511
../data/mizushima_neg/dicom¥14.dcm	0	0.001325
../data/mizushima_neg/dicom¥141.dcm	0	0.0058
../data/mizushima_neg/dicom¥142.dcm	0	0.02246
../data/mizushima_neg/dicom¥149.dcm	0	0.03845
../data/mizushima_neg/dicom¥15.dcm	0	0.001461
../data/mizushima_neg/dicom¥150.dcm	0	0.01484
../data/mizushima_neg/dicom¥151.dcm	0	0.00838
../data/mizushima_neg/dicom¥152.dcm	0	0.000672
../data/mizushima_neg/dicom¥154.dcm	0	0.000833
../data/mizushima_neg/dicom¥155.dcm	0	0.005
../data/mizushima_neg/dicom¥157.dcm	0	0.00067
../data/mizushima_neg/dicom¥158.dcm	0	0.000843
../data/mizushima_neg/dicom¥160.dcm	0	0.002083
../data/mizushima_neg/dicom¥161.dcm	0	0.07104
../data/mizushima_neg/dicom¥162.dcm	0	0.004265
../data/mizushima_neg/dicom¥163.dcm	0	0.011246
../data/mizushima_neg/dicom¥164.dcm	0	0.00582
../data/mizushima_neg/dicom¥165.dcm	0	0.001235
../data/mizushima_neg/dicom¥167.dcm	0	0.002388

../data/mizushima_neg/dicom¥168.dcm	0	0.00259
../data/mizushima_neg/dicom¥17.dcm	0	0.001211
../data/mizushima_neg/dicom¥170.dcm	0	0.002306
../data/mizushima_neg/dicom¥171.dcm	0	0.000701
../data/mizushima_neg/dicom¥172.dcm	0	0.149
../data/mizushima_neg/dicom¥173.dcm	0	0.003136
../data/mizushima_neg/dicom¥174.dcm	0	0.1069
../data/mizushima_neg/dicom¥175.dcm	0	0.3452
../data/mizushima_neg/dicom¥176.dcm	0	0.002958
../data/mizushima_neg/dicom¥177.dcm	0	0.001574
../data/mizushima_neg/dicom¥179.dcm	0	0.001782
../data/mizushima_neg/dicom¥18.dcm	0	0.00435
../data/mizushima_neg/dicom¥180.dcm	0	0.2401
../data/mizushima_neg/dicom¥181.dcm	0	0.667
../data/mizushima_neg/dicom¥182.dcm	0	0.004467
../data/mizushima_neg/dicom¥183.dcm	0	0.001065
../data/mizushima_neg/dicom¥184.dcm	0	0.1334
../data/mizushima_neg/dicom¥185.dcm	0	0.04932
../data/mizushima_neg/dicom¥186.dcm	0	0.3914
../data/mizushima_neg/dicom¥187.dcm	0	0.001536
../data/mizushima_neg/dicom¥188.dcm	0	0.001995
../data/mizushima_neg/dicom¥19.dcm	0	0.000747
../data/mizushima_neg/dicom¥190.dcm	0	0.001099
../data/mizushima_neg/dicom¥191.dcm	0	0.1476
../data/mizushima_neg/dicom¥192.dcm	0	0.0871
../data/mizushima_neg/dicom¥193.dcm	0	0.0156
../data/mizushima_neg/dicom¥195.dcm	0	0.000641
../data/mizushima_neg/dicom¥196.dcm	0	0.002855
../data/mizushima_neg/dicom¥197.dcm	0	0.01942
../data/mizushima_neg/dicom¥198.dcm	0	0.00097
../data/mizushima_neg/dicom¥199.dcm	0	0.002935
../data/mizushima_neg/dicom¥2.dcm	0	0.10913
../data/mizushima_neg/dicom¥200.dcm	0	0.000218
../data/mizushima_neg/dicom¥201.dcm	0	0.003027
../data/mizushima_neg/dicom¥203.dcm	0	0.000446
../data/mizushima_neg/dicom¥204.dcm	0	0.02876
../data/mizushima_neg/dicom¥205.dcm	0	0.000387
../data/mizushima_neg/dicom¥206.dcm	0	0.000566
../data/mizushima_neg/dicom¥207.dcm	0	0.001165
../data/mizushima_neg/dicom¥209.dcm	0	0.000843

../data/mizushima_neg/dicom¥21.dcm	0	0.001574
../data/mizushima_neg/dicom¥211.dcm	0	0.002758
../data/mizushima_neg/dicom¥212.dcm	0	0.0741
../data/mizushima_neg/dicom¥213.dcm	0	0.002378
../data/mizushima_neg/dicom¥215.dcm	0	0.002243
../data/mizushima_neg/dicom¥216.dcm	0	0.00387
../data/mizushima_neg/dicom¥217.dcm	0	0.00598
../data/mizushima_neg/dicom¥218.dcm	0	0.02177
../data/mizushima_neg/dicom¥219.dcm	0	0.00132
../data/mizushima_neg/dicom¥22.dcm	0	0.003136
../data/mizushima_neg/dicom¥220.dcm	0	0.744
../data/mizushima_neg/dicom¥221.dcm	0	0.0636
../data/mizushima_neg/dicom¥222.dcm	0	0.0051
../data/mizushima_neg/dicom¥223.dcm	0	0.004364
../data/mizushima_neg/dicom¥224.dcm	0	0.003063
../data/mizushima_neg/dicom¥226.dcm	0	0.000555
../data/mizushima_neg/dicom¥227.dcm	0	0.00067
../data/mizushima_neg/dicom¥228.dcm	0	0.000911
../data/mizushima_neg/dicom¥229.dcm	0	0.000659
../data/mizushima_neg/dicom¥231.dcm	0	0.000451
../data/mizushima_neg/dicom¥232.dcm	0	0.248
../data/mizushima_neg/dicom¥233.dcm	0	0.004982
../data/mizushima_neg/dicom¥234.dcm	0	0.01567
../data/mizushima_neg/dicom¥235.dcm	0	0.005447
../data/mizushima_neg/dicom¥236.dcm	0	0.002779
../data/mizushima_neg/dicom¥237.dcm	0	0.0196
../data/mizushima_neg/dicom¥24.dcm	0	0.02711
../data/mizushima_neg/dicom¥240.dcm	0	0.001143
../data/mizushima_neg/dicom¥241.dcm	0	0.00433
../data/mizushima_neg/dicom¥242.dcm	0	0.001116
../data/mizushima_neg/dicom¥243.dcm	0	0.001081
../data/mizushima_neg/dicom¥244.dcm	0	0.001032
../data/mizushima_neg/dicom¥245.dcm	0	0.000519
../data/mizushima_neg/dicom¥247.dcm	0	0.002323
../data/mizushima_neg/dicom¥25.dcm	0	0.003325
../data/mizushima_neg/dicom¥250.dcm	0	0.000958
../data/mizushima_neg/dicom¥251.dcm	0	0.5176
../data/mizushima_neg/dicom¥252.dcm	0	0.00819
../data/mizushima_neg/dicom¥253.dcm	0	0.002481
../data/mizushima_neg/dicom¥255.dcm	0	0.001592

../data/mizushima_neg/dicom¥256.dcm	0	0.00452
../data/mizushima_neg/dicom¥257.dcm	0	0.00881
../data/mizushima_neg/dicom¥258.dcm	0	0.004982
../data/mizushima_neg/dicom¥26.dcm	0	0.005344
../data/mizushima_neg/dicom¥260.dcm	0	0.01049
../data/mizushima_neg/dicom¥261.dcm	0	0.001245
../data/mizushima_neg/dicom¥262.dcm	0	0.00982
../data/mizushima_neg/dicom¥264.dcm	0	0.1917
../data/mizushima_neg/dicom¥265.dcm	0	0.003273
../data/mizushima_neg/dicom¥266.dcm	0	0.000609
../data/mizushima_neg/dicom¥269.dcm	0	0.000448
../data/mizushima_neg/dicom¥27.dcm	0	0.000363
../data/mizushima_neg/dicom¥270.dcm	0	0.001095
../data/mizushima_neg/dicom¥271.dcm	0	0.000641
../data/mizushima_neg/dicom¥272.dcm	0	0.000773
../data/mizushima_neg/dicom¥273.dcm	0	0.002867
../data/mizushima_neg/dicom¥274.dcm	0	0.001305
../data/mizushima_neg/dicom¥275.dcm	0	0.002323
../data/mizushima_neg/dicom¥277.dcm	0	0.000817
../data/mizushima_neg/dicom¥278.dcm	0	0.1661
../data/mizushima_neg/dicom¥279.dcm	0	0.002693
../data/mizushima_neg/dicom¥280.dcm	0	0.001285
../data/mizushima_neg/dicom¥281.dcm	0	0.000564
../data/mizushima_neg/dicom¥282.dcm	0	0.00311
../data/mizushima_neg/dicom¥283.dcm	0	0.003248
../data/mizushima_neg/dicom¥284.dcm	0	0.02504
../data/mizushima_neg/dicom¥285.dcm	0	0.002174
../data/mizushima_neg/dicom¥286.dcm	0	0.000721
../data/mizushima_neg/dicom¥287.dcm	0	0.001507
../data/mizushima_neg/dicom¥29.dcm	0	0.02998
../data/mizushima_neg/dicom¥292.dcm	0	0.000494
../data/mizushima_neg/dicom¥293.dcm	0	0.003565
../data/mizushima_neg/dicom¥295.dcm	0	0.0477
../data/mizushima_neg/dicom¥296.dcm	0	0.000883
../data/mizushima_neg/dicom¥299.dcm	0	0.04257
../data/mizushima_neg/dicom¥30.dcm	0	0.000654
../data/mizushima_neg/dicom¥300.dcm	0	0.01519
../data/mizushima_neg/dicom¥301.dcm	0	0.001134
../data/mizushima_neg/dicom¥302.dcm	0	0.000807
../data/mizushima_neg/dicom¥303.dcm	0	0.002216

../data/mizushima_neg/dicom¥304.dcm	0	0.000981
../data/mizushima_neg/dicom¥305.dcm	0	0.000354
../data/mizushima_neg/dicom¥306.dcm	0	0.000406
../data/mizushima_neg/dicom¥307.dcm	0	0.001755
../data/mizushima_neg/dicom¥308.dcm	0	0.000232
../data/mizushima_neg/dicom¥31.dcm	0	0.000811
../data/mizushima_neg/dicom¥311.dcm	0	0.001179
../data/mizushima_neg/dicom¥312.dcm	0	0.009636
../data/mizushima_neg/dicom¥313.dcm	0	0.4805
../data/mizushima_neg/dicom¥314.dcm	0	0.01359
../data/mizushima_neg/dicom¥315.dcm	0	0.002571
../data/mizushima_neg/dicom¥316.dcm	0	0.004906
../data/mizushima_neg/dicom¥317.dcm	0	0.00057
../data/mizushima_neg/dicom¥318.dcm	0	0.6416
../data/mizushima_neg/dicom¥32.dcm	0	0.01927
../data/mizushima_neg/dicom¥322.dcm	0	0.000336
../data/mizushima_neg/dicom¥323.dcm	0	0.00094
../data/mizushima_neg/dicom¥324.dcm	0	0.01302
../data/mizushima_neg/dicom¥325.dcm	0	0.0995
../data/mizushima_neg/dicom¥326.dcm	0	0.00102
../data/mizushima_neg/dicom¥328.dcm	0	0.004314
../data/mizushima_neg/dicom¥329.dcm	0	0.000434
../data/mizushima_neg/dicom¥331.dcm	0	0.000408
../data/mizushima_neg/dicom¥333.dcm	0	0.0882
../data/mizushima_neg/dicom¥334.dcm	0	0.001775
../data/mizushima_neg/dicom¥336.dcm	0	0.02571
../data/mizushima_neg/dicom¥338.dcm	0	0.000458
../data/mizushima_neg/dicom¥339.dcm	0	0.001197
../data/mizushima_neg/dicom¥341.dcm	0	0.001
../data/mizushima_neg/dicom¥342.dcm	0	0.001274
../data/mizushima_neg/dicom¥346.dcm	0	0.000551
../data/mizushima_neg/dicom¥347.dcm	0	0.003286
../data/mizushima_neg/dicom¥348.dcm	0	0.00123
../data/mizushima_neg/dicom¥349.dcm	0	0.000646
../data/mizushima_neg/dicom¥350.dcm	0	0.000776
../data/mizushima_neg/dicom¥351.dcm	0	0.008
../data/mizushima_neg/dicom¥353.dcm	0	0.001138
../data/mizushima_neg/dicom¥354.dcm	0	0.003765
../data/mizushima_neg/dicom¥355.dcm	0	0.000704
../data/mizushima_neg/dicom¥356.dcm	0	0.04385

../data/mizushima_neg/dicom¥357.dcm	0	0.00109
../data/mizushima_neg/dicom¥358.dcm	0	0.01021
../data/mizushima_neg/dicom¥359.dcm	0	0.006565
../data/mizushima_neg/dicom¥361.dcm	0	0.01423
../data/mizushima_neg/dicom¥363.dcm	0	0.009674
../data/mizushima_neg/dicom¥364.dcm	0	0.0009
../data/mizushima_neg/dicom¥366.dcm	0	0.002398
../data/mizushima_neg/dicom¥367.dcm	0	0.009056
../data/mizushima_neg/dicom¥372.dcm	0	0.002003
../data/mizushima_neg/dicom¥373.dcm	0	0.0022
../data/mizushima_neg/dicom¥374.dcm	0	0.001188
../data/mizushima_neg/dicom¥376.dcm	0	0.000562
../data/mizushima_neg/dicom¥377.dcm	0	0.000798
../data/mizushima_neg/dicom¥379.dcm	0	0.000573
../data/mizushima_neg/dicom¥38.dcm	0	0.1807
../data/mizushima_neg/dicom¥380.dcm	0	0.01791
../data/mizushima_neg/dicom¥382.dcm	0	0.000602
../data/mizushima_neg/dicom¥383.dcm	0	0.000627
../data/mizushima_neg/dicom¥385.dcm	0	0.00358
../data/mizushima_neg/dicom¥386.dcm	0	0.00819
../data/mizushima_neg/dicom¥388.dcm	0	0.7324
../data/mizushima_neg/dicom¥389.dcm	0	0.113
../data/mizushima_neg/dicom¥39.dcm	0	0.2319
../data/mizushima_neg/dicom¥390.dcm	0	0.03004
../data/mizushima_neg/dicom¥391.dcm	0	0.01616
../data/mizushima_neg/dicom¥392.dcm	0	0.001265
../data/mizushima_neg/dicom¥393.dcm	0	0.002981
../data/mizushima_neg/dicom¥394.dcm	0	0.001099
../data/mizushima_neg/dicom¥395.dcm	0	0.00109
../data/mizushima_neg/dicom¥396.dcm	0	0.00145
../data/mizushima_neg/dicom¥400.dcm	0	0.00677
../data/mizushima_neg/dicom¥401.dcm	0	0.000907
../data/mizushima_neg/dicom¥402.dcm	0	0.668
../data/mizushima_neg/dicom¥403.dcm	0	0.3154
../data/mizushima_neg/dicom¥404.dcm	0	0.00425
../data/mizushima_neg/dicom¥405.dcm	0	0.00126
../data/mizushima_neg/dicom¥406.dcm	0	0.002758
../data/mizushima_neg/dicom¥407.dcm	0	0.00407
../data/mizushima_neg/dicom¥409.dcm	0	0.00053
../data/mizushima_neg/dicom¥41.dcm	0	0.000499

../data/mizushima_neg/dicom¥411.dcm	0	0.00909
../data/mizushima_neg/dicom¥412.dcm	0	0.001846
../data/mizushima_neg/dicom¥414.dcm	0	0.001057
../data/mizushima_neg/dicom¥416.dcm	0	0.000605
../data/mizushima_neg/dicom¥417.dcm	0	0.001995
../data/mizushima_neg/dicom¥419.dcm	0	0.001151
../data/mizushima_neg/dicom¥42.dcm	0	0.001519
../data/mizushima_neg/dicom¥421.dcm	0	0.00326
../data/mizushima_neg/dicom¥423.dcm	0	0.000482
../data/mizushima_neg/dicom¥424.dcm	0	0.01133
../data/mizushima_neg/dicom¥426.dcm	0	0.0042
../data/mizushima_neg/dicom¥427.dcm	0	0.002369
../data/mizushima_neg/dicom¥429.dcm	0	0.00899
../data/mizushima_neg/dicom¥430.dcm	0	0.00596
../data/mizushima_neg/dicom¥431.dcm	0	0.001315
../data/mizushima_neg/dicom¥433.dcm	0	0.000356
../data/mizushima_neg/dicom¥434.dcm	0	0.001727
../data/mizushima_neg/dicom¥435.dcm	0	0.002314
../data/mizushima_neg/dicom¥437.dcm	0	0.006413
../data/mizushima_neg/dicom¥438.dcm	0	0.000507
../data/mizushima_neg/dicom¥44.dcm	0	0.003052
../data/mizushima_neg/dicom¥440.dcm	0	0.02596
../data/mizushima_neg/dicom¥441.dcm	0	0.007725
../data/mizushima_neg/dicom¥442.dcm	0	0.000985
../data/mizushima_neg/dicom¥444.dcm	0	0.000234
../data/mizushima_neg/dicom¥446.dcm	0	0.0109
../data/mizushima_neg/dicom¥448.dcm	0	0.00181
../data/mizushima_neg/dicom¥449.dcm	0	0.001741
../data/mizushima_neg/dicom¥45.dcm	0	0.000922
../data/mizushima_neg/dicom¥450.dcm	0	0.000598
../data/mizushima_neg/dicom¥451.dcm	0	0.00425
../data/mizushima_neg/dicom¥452.dcm	0	0.001143
../data/mizushima_neg/dicom¥453.dcm	0	0.01423
../data/mizushima_neg/dicom¥454.dcm	0	0.002811
../data/mizushima_neg/dicom¥455.dcm	0	0.0885
../data/mizushima_neg/dicom¥456.dcm	0	0.00573
../data/mizushima_neg/dicom¥458.dcm	0	0.001147
../data/mizushima_neg/dicom¥459.dcm	0	0.000426
../data/mizushima_neg/dicom¥46.dcm	0	0.003325
../data/mizushima_neg/dicom¥460.dcm	0	0.000395

../data/mizushima_neg/dicom¥461.dcm	0	0.0107
../data/mizushima_neg/dicom¥462.dcm	0	0.012924
../data/mizushima_neg/dicom¥464.dcm	0	0.000437
../data/mizushima_neg/dicom¥465.dcm	0	0.000795
../data/mizushima_neg/dicom¥467.dcm	0	0.001305
../data/mizushima_neg/dicom¥468.dcm	0	0.02174
../data/mizushima_neg/dicom¥47.dcm	0	0.000366
../data/mizushima_neg/dicom¥470.dcm	0	0.000843
../data/mizushima_neg/dicom¥471.dcm	0	0.001086
../data/mizushima_neg/dicom¥478.dcm	0	0.002415
../data/mizushima_neg/dicom¥479.dcm	0	0.000755
../data/mizushima_neg/dicom¥480.dcm	0	0.001383
../data/mizushima_neg/dicom¥481.dcm	0	0.001949
../data/mizushima_neg/dicom¥484.dcm	0	0.000272
../data/mizushima_neg/dicom¥485.dcm	0	0.001824
../data/mizushima_neg/dicom¥487.dcm	0	0.000907
../data/mizushima_neg/dicom¥488.dcm	0	0.000451
../data/mizushima_neg/dicom¥489.dcm	0	0.1294
../data/mizushima_neg/dicom¥491.dcm	0	0.010056
../data/mizushima_neg/dicom¥492.dcm	0	0.000636
../data/mizushima_neg/dicom¥493.dcm	0	0.0139
../data/mizushima_neg/dicom¥494.dcm	0	0.003052
../data/mizushima_neg/dicom¥499.dcm	0	0.03528
../data/mizushima_neg/dicom¥5.dcm	0	0.1172
../data/mizushima_neg/dicom¥500.dcm	0	0.000641
../data/mizushima_neg/dicom¥502.dcm	0	0.00919
../data/mizushima_neg/dicom¥504.dcm	0	0.00399
../data/mizushima_neg/dicom¥505.dcm	0	0.000607
../data/mizushima_neg/dicom¥507.dcm	0	0.000427
../data/mizushima_neg/dicom¥508.dcm	0	0.002693
../data/mizushima_neg/dicom¥51.dcm	0	0.000575
../data/mizushima_neg/dicom¥510.dcm	0	0.001057
../data/mizushima_neg/dicom¥511.dcm	0	0.002562
../data/mizushima_neg/dicom¥512.dcm	0	0.002714
../data/mizushima_neg/dicom¥513.dcm	0	0.002958
../data/mizushima_neg/dicom¥514.dcm	0	0.000696
../data/mizushima_neg/dicom¥515.dcm	0	0.000699
../data/mizushima_neg/dicom¥516.dcm	0	0.004925
../data/mizushima_neg/dicom¥518.dcm	0	0.223
../data/mizushima_neg/dicom¥519.dcm	0	0.002935

../data/mizushima_neg/dicom¥52.dcm	0	0.000347
../data/mizushima_neg/dicom¥521.dcm	0	0.000752
../data/mizushima_neg/dicom¥522.dcm	0	0.009125
../data/mizushima_neg/dicom¥524.dcm	0	0.00259
../data/mizushima_neg/dicom¥525.dcm	0	0.01078
../data/mizushima_neg/dicom¥527.dcm	0	0.001107
../data/mizushima_neg/dicom¥528.dcm	0	0.02423
../data/mizushima_neg/dicom¥53.dcm	0	0.002287
../data/mizushima_neg/dicom¥531.dcm	0	0.0039
../data/mizushima_neg/dicom¥532.dcm	0	0.001741
../data/mizushima_neg/dicom¥537.dcm	0	0.003496
../data/mizushima_neg/dicom¥538.dcm	0	0.004005
../data/mizushima_neg/dicom¥539.dcm	0	0.002653
../data/mizushima_neg/dicom¥54.dcm	0	0.001372
../data/mizushima_neg/dicom¥540.dcm	0	0.002611
../data/mizushima_neg/dicom¥541.dcm	0	0.000798
../data/mizushima_neg/dicom¥542.dcm	0	0.00485
../data/mizushima_neg/dicom¥543.dcm	0	0.00644
../data/mizushima_neg/dicom¥544.dcm	0	0.00321
../data/mizushima_neg/dicom¥546.dcm	0	0.001008
../data/mizushima_neg/dicom¥547.dcm	0	0.002058
../data/mizushima_neg/dicom¥549.dcm	0	0.001803
../data/mizushima_neg/dicom¥55.dcm	0	0.001623
../data/mizushima_neg/dicom¥550.dcm	0	0.0052
../data/mizushima_neg/dicom¥551.dcm	0	0.000313
../data/mizushima_neg/dicom¥552.dcm	0	0.002028
../data/mizushima_neg/dicom¥554.dcm	0	0.000492
../data/mizushima_neg/dicom¥555.dcm	0	0.000955
../data/mizushima_neg/dicom¥556.dcm	0	0.005688
../data/mizushima_neg/dicom¥558.dcm	0	0.003052
../data/mizushima_neg/dicom¥559.dcm	0	0.001839
../data/mizushima_neg/dicom¥56.dcm	0	0.003197
../data/mizushima_neg/dicom¥564.dcm	0	0.003075
../data/mizushima_neg/dicom¥565.dcm	0	0.02129
../data/mizushima_neg/dicom¥566.dcm	0	0.000532
../data/mizushima_neg/dicom¥567.dcm	0	0.000553
../data/mizushima_neg/dicom¥569.dcm	0	0.003248
../data/mizushima_neg/dicom¥570.dcm	0	0.01672
../data/mizushima_neg/dicom¥571.dcm	0	0.006718
../data/mizushima_neg/dicom¥572.dcm	0	0.00433

../data/mizushima_neg/dicom¥574.dcm	0	0.003325
../data/mizushima_neg/dicom¥575.dcm	0	0.00934
../data/mizushima_neg/dicom¥577.dcm	0	0.000351
../data/mizushima_neg/dicom¥578.dcm	0	0.006145
../data/mizushima_neg/dicom¥58.dcm	0	0.000798
../data/mizushima_neg/dicom¥580.dcm	0	0.000773
../data/mizushima_neg/dicom¥581.dcm	0	0.00094
../data/mizushima_neg/dicom¥583.dcm	0	0.000612
../data/mizushima_neg/dicom¥584.dcm	0	0.002913
../data/mizushima_neg/dicom¥585.dcm	0	0.000682
../data/mizushima_neg/dicom¥586.dcm	0	0.001078
../data/mizushima_neg/dicom¥588.dcm	0	0.000403
../data/mizushima_neg/dicom¥59.dcm	0	0.001432
../data/mizushima_neg/dicom¥590.dcm	0	0.02307
../data/mizushima_neg/dicom¥591.dcm	0	0.003136
../data/mizushima_neg/dicom¥592.dcm	0	0.001188
../data/mizushima_neg/dicom¥594.dcm	0	0.004887
../data/mizushima_neg/dicom¥595.dcm	0	0.02194
../data/mizushima_neg/dicom¥596.dcm	0	0.002747
../data/mizushima_neg/dicom¥597.dcm	0	0.007755
../data/mizushima_neg/dicom¥598.dcm	0	0.01234
../data/mizushima_neg/dicom¥599.dcm	0	0.004963
../data/mizushima_neg/dicom¥6.dcm	0	0.00149
../data/mizushima_neg/dicom¥60.dcm	0	0.00067
../data/mizushima_neg/dicom¥601.dcm	0	0.01646
../data/mizushima_neg/dicom¥603.dcm	0	0.001668
../data/mizushima_neg/dicom¥604.dcm	0	0.000767
../data/mizushima_neg/dicom¥605.dcm	0	0.04016
../data/mizushima_neg/dicom¥606.dcm	0	0.001032
../data/mizushima_neg/dicom¥607.dcm	0	0.00874
../data/mizushima_neg/dicom¥608.dcm	0	0.002947
../data/mizushima_neg/dicom¥609.dcm	0	0.00068
../data/mizushima_neg/dicom¥610.dcm	0	0.000446
../data/mizushima_neg/dicom¥611.dcm	0	0.001309
../data/mizushima_neg/dicom¥613.dcm	0	0.000431
../data/mizushima_neg/dicom¥614.dcm	0	0.000548
../data/mizushima_neg/dicom¥616.dcm	0	0.02866
../data/mizushima_neg/dicom¥617.dcm	0	0.00479
../data/mizushima_neg/dicom¥619.dcm	0	0.000773
../data/mizushima_neg/dicom¥62.dcm	0	0.0017

../data/mizushima_neg/dicom¥620.dcm	0	0.001065
../data/mizushima_neg/dicom¥622.dcm	0	0.00878
../data/mizushima_neg/dicom¥623.dcm	0	0.004383
../data/mizushima_neg/dicom¥625.dcm	0	0.001796
../data/mizushima_neg/dicom¥626.dcm	0	0.001065
../data/mizushima_neg/dicom¥628.dcm	0	0.001315
../data/mizushima_neg/dicom¥629.dcm	0	0.002306
../data/mizushima_neg/dicom¥63.dcm	0	0.00123
../data/mizushima_neg/dicom¥631.dcm	0	0.00881
../data/mizushima_neg/dicom¥632.dcm	0	0.000755
../data/mizushima_neg/dicom¥636.dcm	0	0.000639
../data/mizushima_neg/dicom¥637.dcm	0	0.001169
../data/mizushima_neg/dicom¥640.dcm	0	0.001561
../data/mizushima_neg/dicom¥641.dcm	0	0.001346
../data/mizushima_neg/dicom¥642.dcm	0	0.006096
../data/mizushima_neg/dicom¥643.dcm	0	0.002758
../data/mizushima_neg/dicom¥645.dcm	0	0.001121
../data/mizushima_neg/dicom¥646.dcm	0	0.011505
../data/mizushima_neg/dicom¥652.dcm	0	0.000301
../data/mizushima_neg/dicom¥653.dcm	0	0.002981
../data/mizushima_neg/dicom¥654.dcm	0	0.003416
../data/mizushima_neg/dicom¥656.dcm	0	0.001942
../data/mizushima_neg/dicom¥658.dcm	0	0.000455
../data/mizushima_neg/dicom¥660.dcm	0	0.1056
../data/mizushima_neg/dicom¥662.dcm	0	0.01001
../data/mizushima_neg/dicom¥663.dcm	0	0.0683
../data/mizushima_neg/dicom¥664.dcm	0	0.000553
../data/mizushima_neg/dicom¥665.dcm	0	0.007725
../data/mizushima_neg/dicom¥666.dcm	0	0.00209
../data/mizushima_neg/dicom¥667.dcm	0	0.001831
../data/mizushima_neg/dicom¥668.dcm	0	0.001755
../data/mizushima_neg/dicom¥669.dcm	0	0.001053
../data/mizushima_neg/dicom¥67.dcm	0	0.002234
../data/mizushima_neg/dicom¥670.dcm	0	0.00214
../data/mizushima_neg/dicom¥673.dcm	0	0.001008
../data/mizushima_neg/dicom¥674.dcm	0	0.00485
../data/mizushima_neg/dicom¥675.dcm	0	0.02849
../data/mizushima_neg/dicom¥676.dcm	0	0.000993
../data/mizushima_neg/dicom¥677.dcm	0	0.003525
../data/mizushima_neg/dicom¥68.dcm	0	0.002684

../data/mizushima_neg/dicom¥7.dcm	0	0.000699
../data/mizushima_neg/dicom¥71.dcm	0	0.00205
../data/mizushima_neg/dicom¥72.dcm	0	0.001221
../data/mizushima_neg/dicom¥73.dcm	0	0.003004
../data/mizushima_neg/dicom¥75.dcm	0	0.0321
../data/mizushima_neg/dicom¥76.dcm	0	0.005665
../data/mizushima_neg/dicom¥77.dcm	0	0.000804
../data/mizushima_neg/dicom¥78.dcm	0	0.001179
../data/mizushima_neg/dicom¥8.dcm	0	0.000455
../data/mizushima_neg/dicom¥80.dcm	0	0.001143
../data/mizushima_neg/dicom¥81.dcm	0	0.009056
../data/mizushima_neg/dicom¥82.dcm	0	0.000504
../data/mizushima_neg/dicom¥83.dcm	0	0.0395
../data/mizushima_neg/dicom¥84.dcm	0	0.001367
../data/mizushima_neg/dicom¥85.dcm	0	0.000744
../data/mizushima_neg/dicom¥86.dcm	0	0.00077
../data/mizushima_neg/dicom¥88.dcm	0	0.002075
../data/mizushima_neg/dicom¥9.dcm	0	0.000704
../data/mizushima_neg/dicom¥90.dcm	0	0.00126
../data/mizushima_neg/dicom¥95.dcm	0	0.004166
../data/mizushima_neg/dicom¥96.dcm	0	0.001525
../data/mizushima_neg/dicom¥97.dcm	0	0.000342
../data/mizushima_neg/dicom¥98.dcm	0	0.02904
../data/mizushima_neg/dicom¥99.dcm	0	0.000747
